# Comparison of bispectral index-guided and fixed-gas concentration techniques in desflurane and remifentanil anesthesia: A randomized controlled trial

**DOI:** 10.1371/journal.pone.0241828

**Published:** 2020-11-05

**Authors:** Seong Mi Yang, Yoo Sun Jung, Chul-Woo Jung, Won Ho Kim, Soo Bin Yoon, Hyung-Chul Lee

**Affiliations:** Department of Anesthesiology and Pain Medicine, Seoul National University College of Medicine, Seoul National University Hospital, Seoul, Republic of Korea; Cleveland Clinic, UNITED STATES

## Abstract

Anesthesia with desflurane and remifentanil can be maintained with either fixed or titrated desflurane concentration. We hypothesized that the fixed-gas concentration (FG) method would reduce the number of anesthetic titrations without hypnotic and hemodynamic instability compared to the bispectral index (BIS)-guided (BG) method. Forty-eight patients were randomly allocated to the FG or BG groups. In the FG group, desflurane vaporizer setting was fixed at 1 age-corrected minimum alveolar concentration (MAC). In the BG group, desflurane was titrated to target a BIS level at 50. Remifentanil was titrated to maintain a systolic arterial pressure (SAP) of 120 mmHg in both groups. Our primary endpoint was the hypnotic stability measured by the wobble of BIS in performance analysis, and the secondary endpoints included the wobble of SAP, mean BIS value during surgery, and the number of anesthetic titrations. The BIS in the FG group showed significantly less wobble (3.9 ± 1.1% *vs* 5.5 ± 1.5%, *P* <0.001) but lower value (33 ± 6 *vs* 46 ± 7, *P* <0.001) than BG group. The wobble of SAP showed no difference between groups [median (inter-quartile range), 5.0 (4.1–7.5)% *vs* 5.2 (4.2–8.3)%, *P* = 0.557]. The numbers of anesthetic titrations in the FG group were significantly lower than the BG group (0 ± 0 vs 8 ± 5, *P*<0.001 for desflurane, 13 ± 13 *vs* 22 ± 17, *P* = 0.047 for remifentanil). Less wobble in BIS and reduced anesthetic titration without hemodynamic instability during the FG technique may be practical in balanced anesthesia using desflurane and remifentanil anesthesia.

**Clinical trial:** This study was registered at ClinicalTrials.gov (NCT02283866).

## Introduction

The current concept of balanced anesthesia is defined as the combined use of a volatile anesthetic agent and an opioid to take advantage of the anesthetic synergy that reduces the use of both drugs [1–4]. This concept of balanced anesthesia can be more refined with the use of EEG-based anesthesia depth monitoring such as bispectral index (BIS). With an EEG monitor, hypnosis, the main effect of the volatile anesthetic agent, can be titrated by an EEG index and the opioid level can be titrated by hemodynamic variables such as systolic arterial pressure (SAP), a traditional surrogate marker of nociceptive stimulation during general anesthesia [5].

However, the nonlinear inverse relationship between volatile anesthetics and opioids makes it difficult to estimate the magnitude of dose adjustment [6]. Titrating one drug inevitably requires adjustment of the other drug. Controlling two synergistic drugs at the same time can lead to hemodynamic or hypnotic instabilities.

The desflurane-remifentanil balanced anesthesia may be more simplified by fixing the desflurane concentration. We hypothesized that the fixed-gas concentration (FG) method can reduce the number of anesthetic titrations with enhanced hemodynamic and hypnotic stabilities compared to the BIS-guided (BG) method. The primary outcome of this study was the stability of the BIS value, to be more exact, the wobble of the BIS value measured by performance analysis [7–10]. The secondary endpoints included the wobble of SAP, mean BIS and SAP value during surgery, total anesthetic drug use, the number of anesthetic titrations, recovery time, and the incidence of explicit recall.

## Materials and methods

After receiving approval from the Seoul National University Hospital Institutional Review Board (1406-098-589), we prospectively studied patients scheduled for elective laparoscopic stomach surgery between September 2014 and January 2015. The current study was registered at ClinicalTrials.gov (NCT02283866; Nov 3, 2014) and was performed in accordance with the relevant guidelines. The authors confirm that all ongoing trials for this intervention are registered. We recruited patients from September 11, 2014 to January 8, 2015. Written informed consent was obtained from the patients during preoperative visits. Patients with convulsive disorder or alcohol abuse; concurrent use of sedative medication, beta blocker or antiarrhythmic agents; severely depressed cardiac function and chronic hypertension; average ward SAP <100 mmHg or >140 mmHg were excluded. Patients were randomized to either the FG or BG group. An unrelated assistant created a randomization in a 1:1 ratio with block randomization technique and the allocation sequence was concealed in sealed envelopes and opened just before anesthesia induction. The participant, investigator, and outcome assessor were all blinded and did not know which group the participant was allocated. Only the anesthesiologist who monitored and anesthetized the patient was informed and performed anesthesia according to the study protocol according to the allocated group.

Noninvasive blood pressure, pulse oximetry, electrocardiography, capnography and BIS were monitored during anesthesia. Blood pressure was measured every 2 minutes. BIS was continuously monitored with BIS VISTA^TM^ monitoring system (Medtronic, Minneapolis, MN, USA). The Primus anesthesia workstation (Dräger Medical, Lübeck, Germany) was used for all of the patients. No premedication was given. Preoxygenation was performed with 100% oxygen and fresh gas flow of 10 L/min. Effect-site target-controlled infusion of remifentanil was started with a target of 3.0 ng/mL using a commercial infusion pump (Orchestra^®^ Base Primea with module DPS, Fresenius Kabi AG, Bad Homburg, Germany) with pharmacokinetic model of Minto [11]. After accomplishing pseudo-equilibrium of remifentanil concentration, propofol 1.2 mg/kg and rocuronium 0.9 mg/kg were administered. During manual ventilation, the desflurane vaporizer setting was set at 1 age-corrected MAC calculated by Mapleson’s equation [12]. Immediately after intubation, the fresh gas flow was reduced to 2 L/min. During maintenance of anesthesia, desflurane concentration was titrated to maintain BIS at 50 by adjusting the vaporizer setting up and down with 1.0 vol% step in the BG group; in the FG group, the vaporizer setting was fixed at 1 age-corrected MAC regardless of the BIS value.

In both groups, the effect site concentration of remifentanil was escalated or decreased with 1 ng/mL step to maintain SAP at 120 mmHg. Sudden changes of SAP more than 30% of previous value needed abrupt changes of remifentanil target as much as 2–3 ng/mL at a time. In this case, the numbers of titrations were counted as 2 and 3, respectively. If the SAP maintained <100 mmHg even at 1 ng/mL effect site concentration of remifentanil, which was predefined as the lower limit of remifentanil use, ephedrine 5 mg was injected intravenously. The maintenance fluid infusion rate during the surgery was 8-10ml/kg/hr. The total infused volume of crystalloids and urine output was measured. If the hourly urine was less than 0.5 mL/kg, 300 mL bolus of crystalloid solution was rapidly infused. Rocuronium was intermittently administered by the guidance of the train-of-four response of the ulnar nerve and the adductor pollicis muscle using the TOF-Watch SX acceleromyography device (Organon, Ireland). The TOF-Watch SX was calibrated before administrating rocuronium during induction of anesthesia. TOF stimulation was continued every 15 s during anesthesia, and when the TOF count was at least 2, rocuronium 5mg was administered.

At the end of surgery, reversal of neuromuscular blockade was done with neostigmine 25 mcg/kg and confirmed with TOF ratio > 0.9. Desflurane and remifentanil administrations were stopped, and fresh gas flow was increased to 10 L/min. The patient was awakened by prodding and calling in a loud tone. Times from the cessation of desflurane and remifentanil administrations to confirmation of observer’s assessment of alertness and sedation (OAA/S) score >2, eye opening, and extubation were recorded [13]. Removal of the endotracheal tube was done immediately after notice of sufficient spontaneous ventilation indicated by negative inspiration pressure <-20 cmH_2_O and measured tidal volume >6 mL/kg.

All the data measured by the patient monitor (Solar^TM^ 8000M, GE healthcare, Milwaukee, WI, USA) including patient’s vital signs and gas concentration were intraoperatively collected from the serial port of the patient monitor using a free data collection program (Vital Recorder v1.7, accessed August 20, 2018, at https://vitaldb.net) [14]. The changes in plasma and effect-site concentrations of remifentanil and BIS were also retrieved with the program. The data were saved at an interval of 2 seconds.

General characteristics of the two groups were retrieved from the electronic medical recording system after surgery, and anesthesia related data collected from the electronic anesthesia record.

The BIS and SAP data during the maintenance period (from 10 minutes after skin incision to 10 minutes before end of surgery) were recorded and analyzed. The mean BIS, HR, and SAP and the incidences of SAP>140mmHg, SAP<100mmHg, and BIS>60 were calculated. The hemodynamic and hypnotic stabilities were evaluated with the performance analysis [7]. The original formulae of performance analysis were proposed to measure the discrepancy between the predicted and measured drug concentrations, however they have been applied to test the variability of SAP and BIS during anesthesia [8–10]. We also adopted the measurements in the performance analysis, such as median performance error (MDPE), median absolute performance error (MDAPE) and wobble [15] to test the bias, error, and the stabilities of BIS and SAP. MDPE stands for the percent bias of the measured values from the predefined target value. MDAPE represents the absolute percent error of the measured values from the target value. Wobble is an index of the percent fluctuation in the performance error. The targeted values in the current study are 120 mmHg for SAP and 50 for BIS throughout the anesthesia maintenance.

The performance measures were calculated by the following equations.

$${\mathrm{PE}}_{ij}{}_{}=\left(Measured_{ij}–Target_{ij}\right)\;/\;Target_{ij}x\;100\%$$

$${\mathrm{MDPE}}_{i}=median\left(PE_{ij}\right)$$

$${\mathrm{MDAPE}}_{i}=median\left(\left|PE_{ij}\right|\right)$$

$${\mathrm{Wobble}}_{i}=median\left(\left|PE_{ij}-MDPE_{i}\right|\right)$$

where *ij* denotes the *j*th measurement in the *i*th subject.

The number of desflurane and remifentanil titrations was counted from the line plots illustrating changes in end-tidal concentrations of desflurane and target effect-site concentrations of remifentanil during the study period. The total dose of desflurane as liquid was calculated with the following formula [16].

$$\text{Total}\;\text{dose}\;\text{as}\;\text{liquid}\;\left(\text{ml}\right)=\frac{\text{mean}\;\text{concentration}\;\text{(vol\%)}\times \;\text{(ml/min)}\times \text{total}\;\text{duration}\;(\text{min})}{\text{210}\;(\text{ml/ml})}$$

where the mean concentration was the time-averaged vaporizer setting.

The incidence of explicit recall of memory was investigated using a questionnaire during the postoperative visit.

### Sample size calculation and statistical analyses

We assumed that the wobble of BIS would be significantly less in the FG group than the BG group. Assuming a mean difference of 8% with an SD of 10%, which was derived from our pilot data, we calculated that 21 patients per each group would be needed with a two-sided alpha error of 0.05 and power of 0.8 using G*power 3.1 [17]. Assuming a drop-out rate of 10%, 24 patients per group were required.

According to the Consolidated Standards of Reporting Trials standards for randomized clinical trials, the intention to treat analysis was done. An investigator blinded to the group assignment reviewed the electronic medical record and analyzed the vital signs data. Assuming that the missing values were missing at random, multiple imputation was used to generate a number of datasets equal to the percentage of missing cases. These datasets were separately analyzed to produce pooled statistical results.

The primary outcome was compared using student T-test or Mann-Whitney U-test based on the result of Kolmogorov-Smirnov test. The secondary outcomes such as MDPE and MDAPE of BIS, performance measures of SAP, the incidence of out-of-range values, and the number of anesthetic titrations were also compared with student T-test or Mann-Whitney U-test based on the result of Kolmogorov-Smirnov test. The incidence of explicit recall was compared with Pearson chi-square test. Kaplan-Meier survival analysis with log rank test for pairwise comparison was performed to compare recovery times defined by OAA/S score >2, eye opening and extubation between two groups.

All data are expressed as mean ± SD, median (interquartile range) or absolute numbers (percentage). A *P*-value <0.05 was considered statistically significant. Statistical analyses were done with SPSS (IBM SPSS Statistics for Windows, version 23.0; IBM, Armonk, NY, USA).

## Results

**Fig 1:**
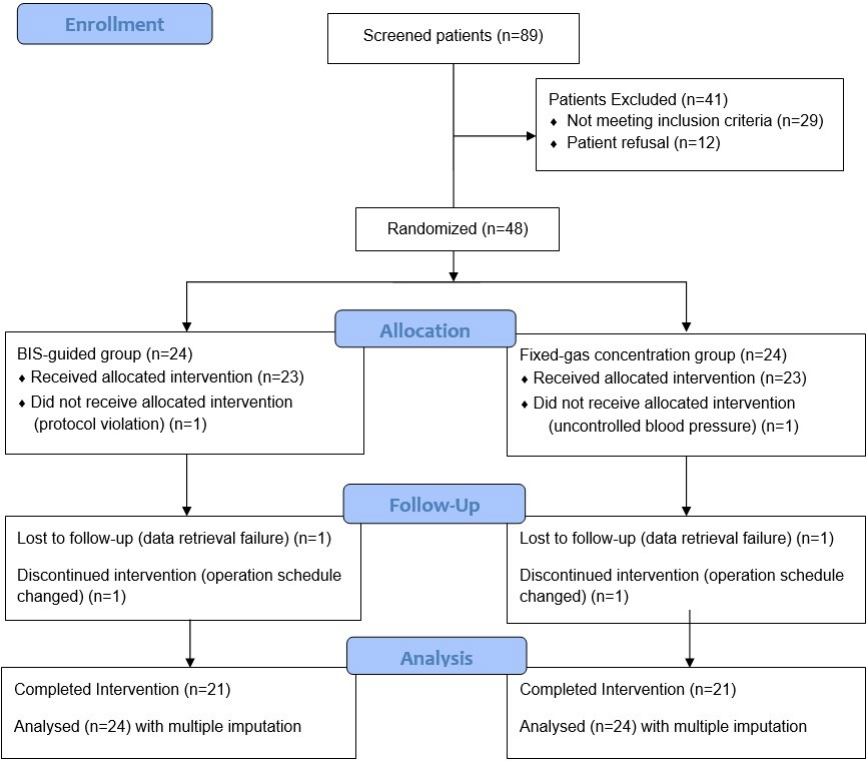
CONSORT diagram.

Eligibility was tested in 89 patients scheduled for laparoscopic stomach surgery: 41 patients were excluded (29 patients met the exclusion criteria and 12 patients refused to participate in the study). A total of 48 patients were recruited and randomized into either the FG group (n = 24) or the BG group (n = 24) and written informed consents were taken. One patient in the BG group had a protocol violation and one patient in the FG group who had uncontrolled blood pressure did not receive the allocated intervention. Two patients from each group were lost to follow-up due to change of surgical plan (*n* = 2) and technical failure to retrieve data (*n* = 2). The missing data for these 6 patients were imputed by multiple imputation for the ITT analysis. All 48 patients (*n* = 24 for each group) entered into analysis (Fig 1).

The general characteristics and variables related to surgery and anesthesia are summarized in Table 1.

**Table 1:** General characteristics and comparison of variables related with desflurane and remifentanil use.

	BIS-guided group (n = 24)	Fixed-gas concentration group (n = 24)
Sex (M/F)	10/14	14/10
Age (years)	57 ± 10	56 ± 11
Weight (kg)	58.9 ± 8.6	62.6 ± 9.7
BMI (kg·m^-2^)	23.1 ± 3.4	23.1 ± 2.6
Duration of surgery (min)	189± 60	171 ± 50
Duration of anesthesia (min)	230 ± 72	209 ± 51
Administered fluid (mL)	1668 ± 716	1498 ± 566
Urine output (mL)	152 ± 80	138 ± 91
Estimated blood loss (mL)	94 (30–150)	50 (30–100)

Values are numbers, mean ± SD or median (interquartile range).

**Fig 2:**
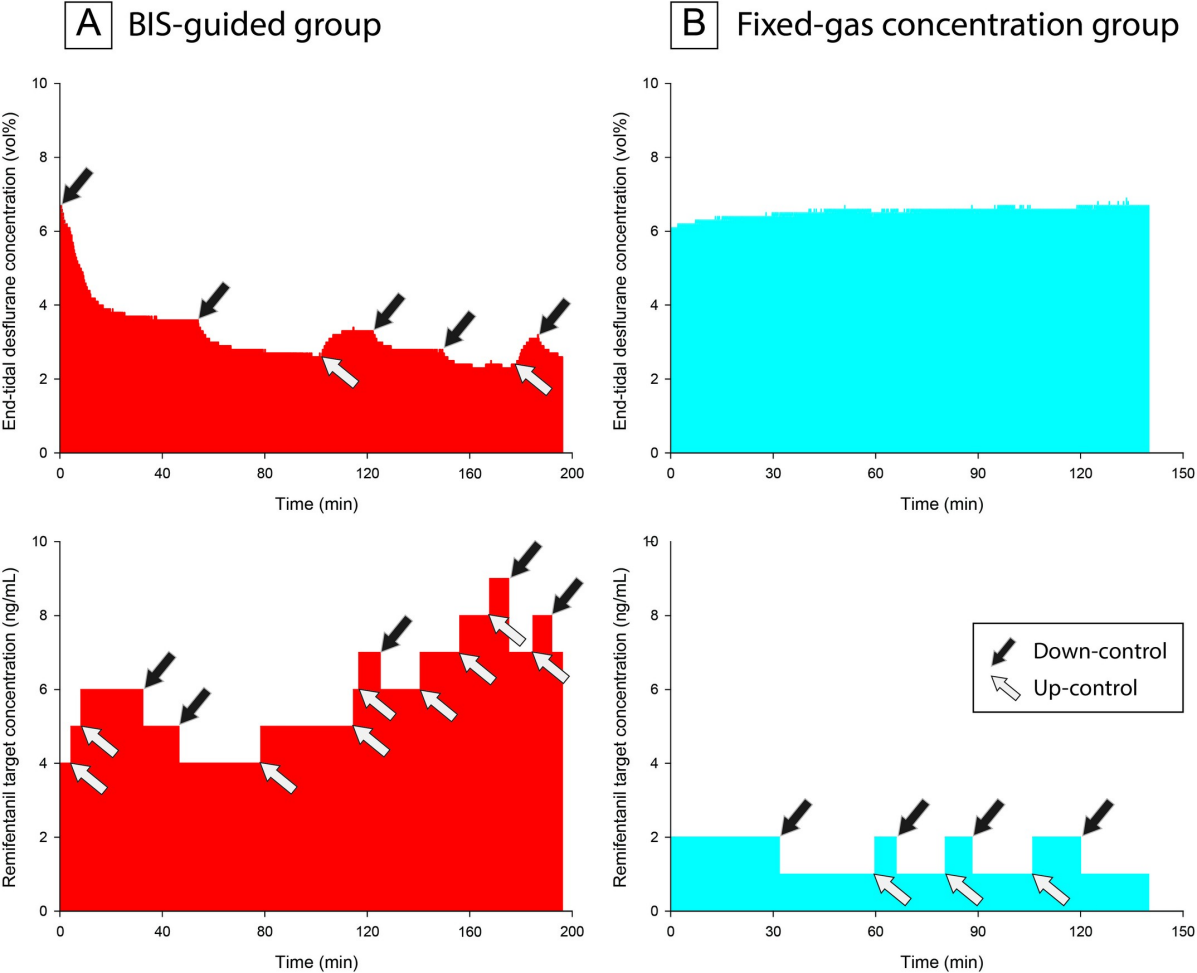
Changes of desflurane and remifentanil target concentrations during balanced anesthesia in typical patients. A: the 7th patient in the bispectral index-guided group; B: the 12th patient in the fixed-gas concentration group. End-tidal concentrations of desflurane and target effect-site concentrations of remifentanil changed during the study period, showing significant difference in the number of titrations between two groups.

Performance analysis of BIS showed that the FG group was related with significantly less wobble (3.9 ± 1.1% *vs* 5.5 ± 1.5%, *P*<0.001). However, the FG group showed significantly lower MDPE (-34.1 ± 11.9% *vs* -8.7 ± 13.3%, *P*<0.001) and greater MDAPE (34.3 ± 11.4% *vs* 11.4 ± 13.2%, *P*<0.001) than the BG group (Table 2). Performance analysis of SAP showed no significant difference between groups. Incidence of SAP <100 mmHg or >140 mmHg was similar between groups. Event of BIS >60 was only found in the BG group. However, the incidence of BIS >60 was less than 1% of entire study period and median time of 90 s. Total dose and mean end tidal concentration of desflurane were significantly lower in the BG group; total dose and mean effect site concentration of remifentanil were significantly lower in the FG group. The numbers of anesthetic titrations in the FG group were significantly lower than the BG group (0 ± 0 vs 8 ± 5, *P* = <0.001 for desflurane, 13 ± 13 *vs* 22 ± 17, *P* = 0.047 for remifentanil, respectively, Table 2 and Fig 2).

**Table 2:** Comparison of hypnotic and hemodynamic stabilities, anesthetic drug uses and recovery time between two groups.

		BIS-guided group (n = 24)	Fixed-gas concentration group (n = 24)	*P-value*
**Primary Outcome**				
Performance of BIS control				
	Wobble (%)	5.5 ± 1.5	3.9 ± 1.1	<0.001
**Secondary Outcomes**				
Performance of BIS control				
	MDPE (%)	-8.7 ± 13.3	-34.1 ± 11.9	<0.001
	MDAPE (%)	11.4 ± 13.2	34.3 ± 11.4	<0.001
Performance of SAP control				
	MDPE (%)	-2.0 ± 6.2	-5.6 ± 4.5	0.050
	MDAPE (%)	8.5 ± 4.1	9.2 ± 3.5	0.576
	Wobble (%)	5.2 (4.2–8.3)	5.0 (4.1–7.5)	0.557
BIS, HR, and SAP during surgery				
	BIS	46 ± 7	33 ± 6	<0.001
	HR (bpm)	67 ± 13	68 ± 10	0.988
	SAP (mmHg)	120 ± 8	115 ± 6	0.400
Incidence of out-of-range value (%)				
	SAP >140 mmHg	9.5 ± 8.4	5.5 ± 4.9	0.097
	SAP <100 mmHg	7.6 ± 5.9	8.6 ± 7.7	0.734
	BIS >60	2.0 ± 2.4	0	0.001
Desflurane use				
	Total dose as liquid (mL)	66.2 ± 34.0	91.5 ± 32.4	0.013
	End-tidal concentrations (age-corrected MAC)			
	Initial	0.6 ± 0.2	0.8 ± 0.1	0.001
	Final	0.6 ± 0.2	0.9 ± 0.2	<0.001
	Average	0.6 ± 0.2	0.9 ± 0.1	<0.001
	Number of titrations	8 ± 5	0 ± 0	<0.001
Remifentanil use				
	Total dose (mcg)	1714 ± 1103	897 ± 428	0.001
	Target effect-site concentrations (ng/mL)			
	Initial	3 (2–3.9)	2 (1–4)	0.492
	Final	4 (2–6)	1 (1–3)	0.041
	Average	3.6 (2.1–5.6)	2 (1–3)	0.001
	Number of titrations	22 ± 17	13 ± 13	0.047
Recovery time				
	OAA/S score	243 ± 152	254 ± 97	0.896
	Eye-opening	323 ± 235	356 ± 122	0.598
	Extubation	383 ± 299	381 ± 165	0.988
Explicit recall		0 (0%)	0 (0%)	1.000

Values are numbers, mean ± SD or median (interquartile range). T-test were used for comparison of two groups. The anesthetics use data were retrieved from 10 min after skin incision to 10 min before the end of surgery. Abbreviations: BIS = bispectral index; MDPE = median performance error; MDAPE = median absolute performance error; SAP = systolic arterial pressure; HR = heart rate; MAC = minimum alveolar concentration; OAA/S = observer’s assessment of alertness/sedation

**Fig 3:**
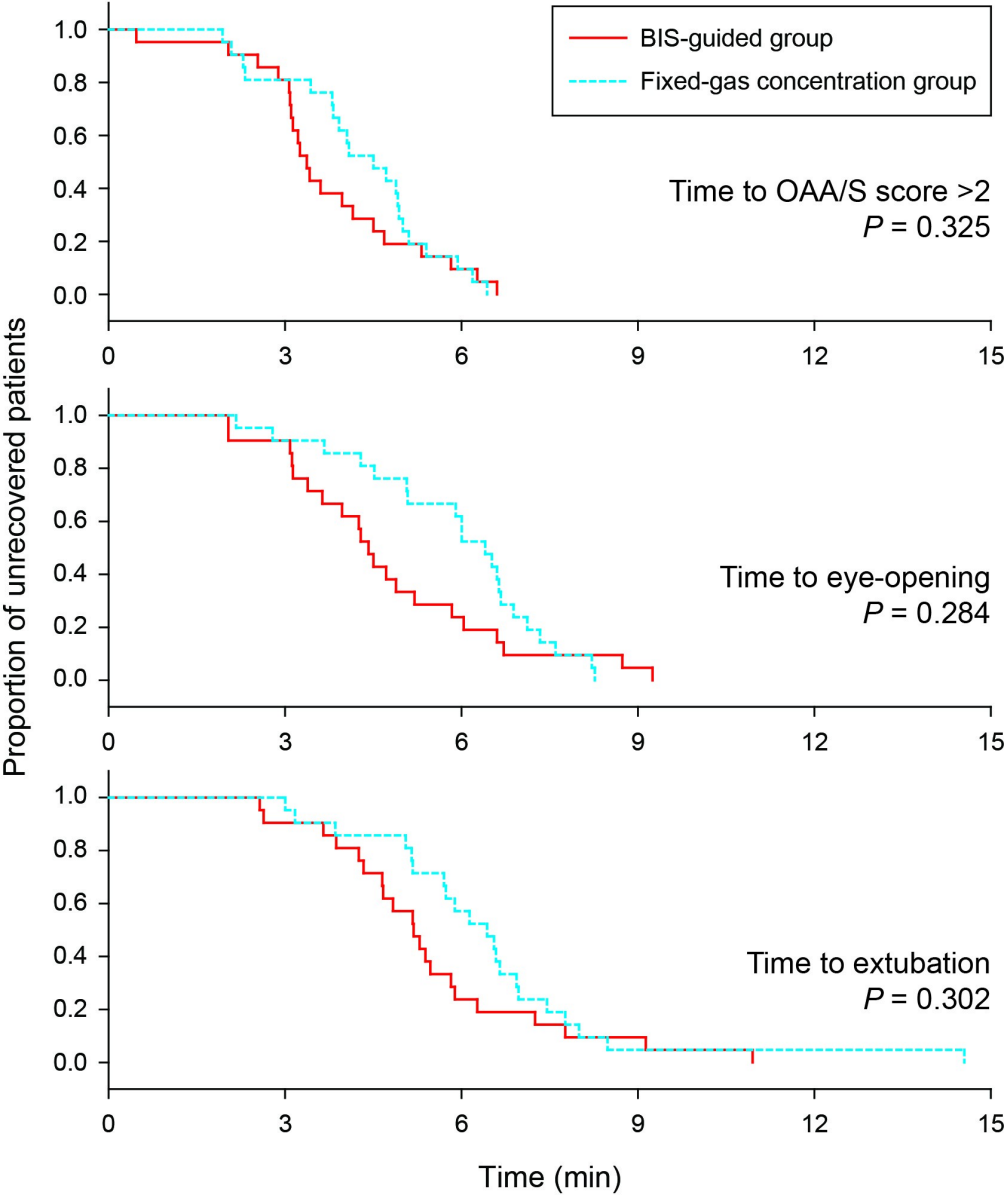
Recovery times defined by different measures like OAA/S score, eye-opening and extubation. Recovery time was defined as the elapsed time between cessation of desflurane and remifentanil and occurrence of recovery signs like OAA/S score >2, opening of eyes to verbal command, or spontaneous ventilation. Extubation was performed immediately after the notice of spontaneous ventilation and means final recovery in this study. Kaplan-Meier survival analysis with log rank test for pairwise comparison reveals that there are no between-group differences regarding three recovery times. Abbreviation: OAA/S = observer’s assessment of alertness/sedation.

Recovery times assessed with OAA/S score >2, eye opening and extubation were similar between groups (Table 2). Kaplan-Meier curves showed that there was no statistical difference in recovery times between two groups (Fig 3).

Postoperative visits revealed no evidence of explicit recall of memory in any patients.

## Discussion

In this study comparing the BG with FG techniques using desflurane and remifentanil for balanced anesthesia, the FG group showed more stable but lower BIS value with fewer number of anesthetic titrations compared to the BG group. Both balanced anesthesia techniques showed stable SAP. Recovery time was also similar between the two groups.

In balanced anesthesia, the hypnotic level and control of nociceptive response are usually titrated according to the BIS level and blood pressure, respectively. However, because the two drugs have a synergistic effect, adjusting one drug can lead to the adjustment of the other drug, which is often repeated many times during surgery. Using the BG technique, for example, if the desflurane vaporizer concentration is increased due to a rise in BIS, the blood pressure as well as BIS may decrease. Decreased blood pressure will call for a lowering level of remifentanil that may result in inadequate control of nociceptive response and an increase of BIS, which will in turn require more desflurane. The whole sequence will be repeated when strict control of both BIS and blood pressure is attempted. A typical example showing an increase in remifentanil control with changes in desflurane target is illustrated in the BG group in Fig 2. Adjustment of remifentanil occurred twice as frequently as that of desflurane. Large inter-individual variability, inaccuracy of vaporizers and inconvenience of frequent need of adjustments may make this *theoretically ideal* BIS-guided balanced anesthesia technique less practical in routine anesthesia practice.

**Fig 4:**
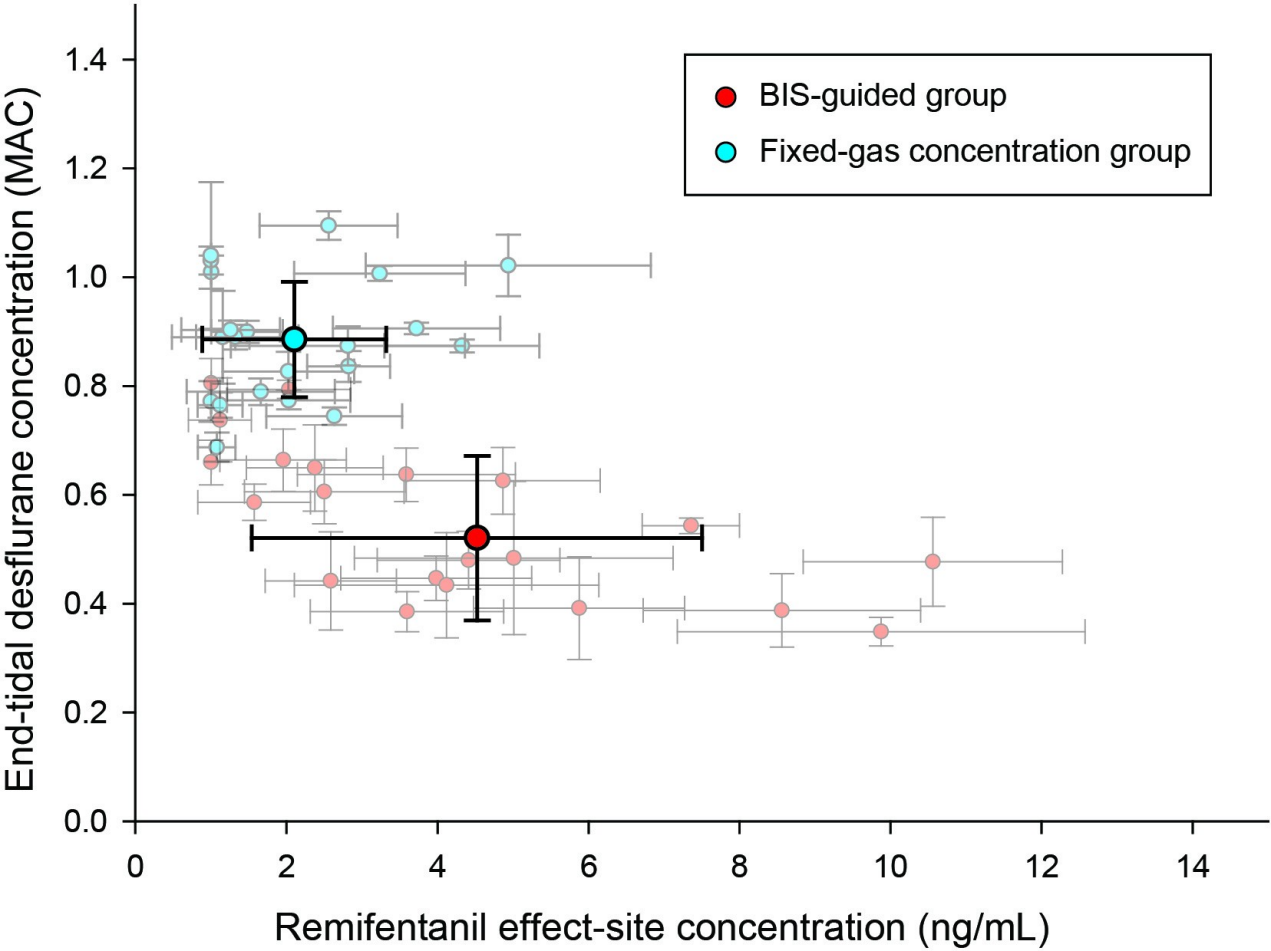
Desflurane-remifentanil concentration combination. Scatter plots represent average (dot) and SD (error bars) of desflurane-remifentanil concentration combinations. The bidirectional error bars represent the variability of desflurane (vertical error bar) and remifentanil (horizontal error bar) concentrations. The fixed-gas concentration group shows higher desflurane and lower remifentanil concentrations with shorter error bars than the bispectral index-guided group.

In the current study, the FG technique was proposed to reduce the number of anesthetic titrations with enhanced hypnotic and hemodynamic stabilities. In this technique, desflurane was the fixed drug because it has a slower equilibration time compared to remifentanil (3.8 min vs 1.8 min) [18, 19]. Remifentanil was the drug to adjust due to its prompt action as well as easy controllability using the target-controlled infusion. According to the results of this study, FG technique showed less instability in BIS value with smaller number of titrations. The FG technique has some advantages over the BG technique. According to the results of this study, it can reduce the number of remifentanil and desflurane dose adjustments. Reduced workload of anesthesiologists can allow more time to focus on patient care. Also, the narrow target range and small variance of remifentanil in the FG group (1–3 ng/mL, Fig 4) may enable the use of simple continuous infusion instead of the target-controlled infusion. However, it must be emphasized that these results of this study cannot be generalized when using different flow rates and different inhalation anesthetics. Further studies should be performed to conclude the benefits and harms of the fixed-gas concentration method especially for more soluble agents, such as isoflurane or halothane.

The total dose of remifentanil was significantly higher in the BG group (Table 2 and Fig 4). In order to maintain a BIS value of 50, the anesthesiologist lowers the vaporizer setting concentration, which is also shown by the significantly lower total desflurane use in the BG group. This results in a rise in the SAP of the patients leading to an increase the remifentanil target concentration.

The SAP of the FG group was well controlled at the target level with just slightly lower MDPE than that of the BG group without a significant difference (-5.6% *vs* -2.0%, 4 mmHg difference, *P* = 0.050). MDAPE was similar in both groups, with the value of 9% (11 mmHg, *P* = 0.576). Wobble of SAP was only 5% (6 mmHg) in both groups. Therefore, we may claim that both balanced anesthesia techniques are successful in maintaining hemodynamic stability.

The lower BIS value in FG group raises concerns about the deep hypnosis related side effects. The mean BIS value of 33 in FG group is obviously below the lower limit of generally recommended surgical anesthesia level. In a previous retrospective study, Monk and colleagues reported that the intraoperative deep hypnotic time (BIS <45) was a major risk factor of 1-year mortality [20]. Leslie and colleagues also reported that intraoperative BIS under 40 for more than 5 minutes increased morbidity and mortality [21]. However, the available evidence on anesthetic depth and long-term survival is inconclusive [22, 23]. Moreover, further lowering the fixed vaporizer setting of the anesthetic agent may increase the risk of awareness. This target concentration of the FG group was determined after considering 0.7 MAC to prevent awareness [24, 25], the 15% margin of error for Tec 6 vaporizer [26], and the 0.8% equilibration ratio of the inspired (F_I_) and expired (F_E_) desflurane [19]. Future studies to find optimal fixed vaporizer setting and to investigate the long-term outcome of FG technique are required.

This study has several limitations. First, the vaporizer setting, not the end-tidal concentration of desflurane was used in the FG group. This was because we expected that the fixed vaporizer setting can be used more conveniently than end-tidal concentration-guided control. Second, the vaporizer setting of 1 age-corrected MAC used in the FG group might have been an overdose, leading to different levels of hypnosis in both groups, especially deep hypnosis in the FG group. Therefore, the advantages in the FG technique such as enhanced hypnotic stabilities with lower number of titrations might be a result of the deep level of anesthesia itself. Third, although there are still controversies, some large studies have reported the detrimental effects of low BIS during general anesthesia [23, 27, 28]. Therefore, further researches investigating the safety of low BIS in FG group and optimizing gas concentration for the FG technique is required. Forth, we may not conclude that the increased number of titrations in BG group actually causes a fatigue in anesthesia personnel because the practitioner’s workload was not measured by a validated tool such as NASA-TLX [29]. Rather, this level of activity might keep the anesthesiologist more vigilant and engaged with the case. Fifth, setting the desflurane vaporizer at 1 age-corrected MAC during manual ventilation and maintaining the fresh gas flow at 2L/min during maintenance may not be the best possible ways to perform anesthesia with desflurane. Desflurane has polluting effects, and the need for using desflurane during induction is questionable [30]. Finally, the benefits of the FG technique cannot be concluded with the use of other flow rates and inhalational agents.

In conclusion, the FG technique using a fixed vaporizer setting of 1 age-corrected MAC desflurane can reduce hypnotic instability and the number of the anesthetic titrations without hemodynamic instability during remifentanil and desflurane anesthesia. The FG technique can be considered a practical method of balanced anesthesia due to its simplicity, convenience, and stable anesthesia delivery. However, future studies are needed to confirm the safety of deep hypnosis associated with the FG technique, to determine the optimal fixed vaporizer setting, and to clarify the effects of other flow rates and other volatile anesthetics.

## Supporting information

S1 File CONSORT 2010 checklist. (DOC)

S2 File Study protocol. (DOCX)

S3 File The anonymized dataset of the current study. Column description: OPTIME = Operation Time (min); ANESTIME = Anesthesia Time (min); EXPTIME = Maintenance time from 10 minutes after skin incision to 10 minutes before end of surgery (min); UO = Urine output (mL); EBL = Estimated blood loss (mL); TOTAL_REMI = Total remifentanil dose (mcg); TOTAL_DES = Total desflurane dose (mL); RFTN_INITIAL = Initial target effect-site concentration (ng/mL) of remifentanil; RFTN_FINAL = Final target effect-site concentration (ng/mL) of remifentanil; RFTN_MEAN = Mean target effect-site concentration (ng/mL) of remifentanil; HighBIS = Frequency of BIS>60 (%); BIS_MDPE = median performance error (%) of bispectral index; BIS_MDAPE = median absolute performance error (%) of bispectral index; BIS_WOBBLE = wobble (%) of bispectral index; SAP_MDPE = median performance error (%) of systolic arterial pressure; SAP_MDAPE = median absolute performance error (%) of systolic arterial pressure; SAP_WOBBLE = wobble (%) of systolic arterial pressure; BIS_MEAN = mean bispectral index value; HR_MEAN = mean heart rate; SBP_MEAN = mean systolic arterial pressure; REMI_CTRL = number of remifentanil titration; DES_CTRL = number of desflurane titration; OAAS_TIME = recovery time assessed with Observer's Assessment of Alertness/Sedation Scale (OAA/S); EO_TIME = recovery time assessed with eye opening; EXTU_TIME = recovery time assessed with extubation; AWARENESS = explicit recall.
(CSV)

S1 Material (ZIP)
