# Supplementary material for: Comparison of bispectral index-guided and fixed-gas concentration techniques in desflurane and remifentanil anesthesia: A randomized controlled trial
Source: PLoS One. 2020 Nov 5;15(11):e0241828. doi: 10.1371/journal.pone.0241828 (PMC7644031; doi:10.1371/journal.pone.0241828)
Supplement: S2 File — (DOCX) [file pone.0241828.s002.docx]

**Bispectral Index-Guided versus Fixed Dose Administration of Desflurane during Balanced Anesthesia with Remifentanil**

**Version No: 1.2**

**Department of Anesthesiology & Pain Medicine**

**Principal Investigator: Chul-Woo Jung**

| Title | Bispectral Index-Guided versus Fixed Dose Administration of Desflurane during Balanced Anesthesia with Remifentanil |
| --- | --- |
| Principal Investigator | Chul-Woo Jung |
| Purpose | Balanced anesthesia is the combined use of a volatile agent and an opioid. The effects of adjusting the desflurane amount targeting a Bispectral Index of 50 and fixing the desflurane dose at 1 MAC (Minum Alveolar Concentration) in patients undergoing balanced anesthesia using desflurane as the inhalational agent and remifentanil as an opioid were compared. |
| Study design | Randomized Controlled Trial |
| Period | IRB approval ~ 12 months |
| Subjects | Patients undergoing laparoscopic gastrectomy under general anesthesia |
| Total number of patients | 48 |
| Vulnerable subjects | Not included |
| Methods | Balanced anesthesia with desflurane and remifentanil is performed. Depending on the randomly assigned group, one group controls the end-tidal concentration of desflurane with the goal of maintaining a BIS value of 50 (range 40-60), while the other group maintains a fixed dose of desflurane at 1 MAC. Remifentanil administration aims to maintain systolic blood pressure at 120mmHg (range 100-140mmHg), apart from desflurane. |
| Effectiveness Evaluation | The BIS values and the stability of vital signs between the two groups during anesthesia maintenance were evaluated. The time from the end of surgery to awakening were compared |
| Safety Evaluation | All patients participating in this study should be observed and evaluated for abnormal BIS values abnormal vital signs. The occurrence of arousal during surgery is evaluated. |
| Expected Results | Among the methods of balanced anesthesia, the sleep state during anesthesia and vital signs will be more stable in the group in which the concentration of inhalation anesthetic is fixed at 1 MAC, which is a clinically more convenient method than in the theoretically superior method of controlling desflurane by the BIS value. |

1. **Background and Purpose**

**1) Background**

In modern anesthesia, 'balanced anesthesia' refers to the method of general anesthesia using an appropriate amount of opiate in combination with an inhalation anesthetic. This method has the advantage of reducing the side effects of excessive use of inhalation anesthetics, namely hemodynamic instability, delay of awakening time, etc. compared to the case of using only inhalation anesthetics by using an appropriate amount of opiate analogs. However, excessive administration of opiate analogs or excessively low inhalation anesthetics may cause problems such as hypotension, bradycardia, or intraoperative arousal. It is important to balance the dose of opiate-like and inhalation anesthetics in order to increase the expected effect and reduce side effects in performing balanced anesthesia.

The 'balance' in balanced anesthesia is theoretically possible through any combination on the same effect curve of inhalation anesthetics and opiate analogs. According to Manyam et al., it is recommended to use an appropriate amount of opiate analog that minimizes and aids inhalation anesthesia, as this ensures the fastest awakening time with a stable hemodynamic condition. For anesthetic equilibrium between sevoflurane and remifentanil, the corresponding concentration is between 0.35-0.5 MAC and 5-7 ng / ml for remifentanil. However, the use of such low concentrations of inhalation anesthesia may cause arousal during surgery due to the change of stimulus during surgery. To minimize the use of inhalation anesthesia, the patient's alertness is monitored using BIS and so on. It is necessary to adjust and use inhalation anesthetics aiming at a BIS of 60. At this time, remifentanil needs to be adjusted according to the hemodynamic status separately from inhalation anesthetics.

However, in reality is many operating theaters cannot routinely use the BIS monitor to apply this theoretical method in clinical practice. A more common and practical method is the fixed administration of inhalation anesthetics above a minimum concentration without the possibility of arousal. Avidan et al. reported a minimal concentration of 0.7-1.3 MAC for inhalation anesthesia without BIS monitoring, and reported that BIS 40-60 remained stable at an average end-tidal concentration of 0.8 MAC. Therefore, a stable and practical balanced anesthesia method that can be performed without BIS monitoring may be a method of fixing inhalation anesthetic administration with an end-tidal concentration of 0.8 MAC and adjusting the dose of remifentanil for hemodynamic stability.

**2) Purpose**

In this study, we compare two methods of controlling inhalation anesthetics under balanced anesthesia. The BIS-guided technique has a theoretically good background with a BIS value of 40-60. This method can greatly reduce the use of inhalation anesthetics, which is advantageous in terms of postoperative arousal speed and hemodynamic stability can be achieved with sufficient opiate analogues, however adjusting the dose of the inhalational agent and opiate analogues from time to time may result in a lack of stable anesthesia or serious fatigue of the anesthesiologist. Inhalation anesthesia is administered at a fixed dose of 1 MAC (in this case, the end-tidal concentration is about 0.8 MAC), and the use of opiate analogs to control hemodynamic status is a practical and easy to use clinical practice. It can be pointed out that low blood pressure and delayed postoperative awakening may occur due to the use of relatively high concentrations of inhaled anesthetics. Compared to the above, there is an advantage that more stable BIS value and maintenance of biological signs are possible. The purpose of this study is to compare the two methods in terms of clinical efficacy and stability and to show the benefits of a practical fixed dose technique.

1. **Selection Criteria, Exclusion Criteria, Target Number of Subjects and Basis for Calculation**
2. **Selection Criteria**

Patients undergoing laparoscopic gastrectomy under general anesthesia for regular surgery and who are indicated for balanced anesthesia using inhaled anesthetic desflurane and opiate analog remifentanil.

Patients with an average systolic blood pressure measured in the ward of 100-140 mmHg

1. **Exclusion Criteria**

Patients not agreeing to the study

Patients undergoing general anesthesia other than balanced anesthesia using inhaled anesthetic desflurane and opiate analog remifentanil

Patients with allergies to neuromuscular blockers, anesthetics, opiates

Patients with a history of malignant hyperthermia

Patients taking CNS agonists

Chronic alcoholics

Patients being treated for paroxysmal disease

Severe cardiac dysfunction patients (EF <30%)

Patients with American Society of Anesthesiologists Grade IV or higher

Patients with systolic blood pressure below 90 mmHg before starting anesthesia

Patients requiring sustained infusion of vasopressors due to hemodynamic instability during surgery

Patients with anesthesia time less than 30 minutes

Patients with chronic hypertension or ischemic heart disease

Patients with cerebral ischemic disease

Patients taking beta blockers or antiarrhythmics

1. **Target Number of Subjects and Basis for Calculation**

After IRB approval, 21 patients in each group are to be collected and analyzed. Given the 10% dropout rate, 24 patients will be recruited for each group.

The primary goal in this study is to compare the difference in 'stability of anesthesia' between the two groups in BIS and systolic blood pressure, respectively, to demonstrate the superiority of the fixed dose technique. However, due to the intrinsic characteristics of balanced anesthesia, the difference in systolic blood pressure is thought to be small, and the main difference between the two groups is expected to be the difference in stability of BIS values. The stability of BIS is expressed as wobble (lower the value the more stable) among the variables of PM (described below in the statistical method part). The BIS records (1 minute interval data automatically stored in the internal memory of the device) from 4 and 3 patients who received balanced anesthesia similarly as the BIS guided and fixed dose group, respectively were analyzed. The MDPE from the calculation of PM from BIS was -3.2 +/- 5.6% and -24 +/- 11.2%, respectively. The average wobble was 10% and 6% (5% standard deviation). Accordingly, when the number of subjects required in each group was calculated assuming one-tailed, type I error (α) 0.05, and power 0.8, each group needed a maximum of 21 patients in the evaluation of wobble. Assumed a dropout rate of 10%, a total of 48 patients, 24 in each group, are expected.

1. **Recruitment Plan**

Patients between the ages of 20 and 80 who are scheduled to undergo surgery under general anesthesia will be given written consent by the investigator after explanation to the patient in accordance with the accompanying instructions and consent. Principal researchers and medical institutions in this study will not exclude patients who are likely to participate in this study based solely on race or socioeconomic status.

1. **Methods**
2. **Specific Research Methods**

The study is performed on patients undergoing laparoscopic gastrectomy under general anesthesia. Prior to induction of anesthesia, patients are divided into BIS-guided group and fixed-dose group in a randomized order.

Anesthesia induction method is the same in both groups. When the patient arrives at the operating room, electrocardiogram, pulse oximeter, non-invasive blood pressure monitor, and BIS monitor are attached and the patient is oxygenated with 100% oxygen. Induction of general anesthesia is performed using propofol and opiate remifentanil. After stabilization of the patient's condition, remifentanil is injected with a target concentration of 5ng / ml using a target concentration control injection method using the Minto model. When the target concentration is reached, 1.2mg/kg propofol is injected. After 30 seconds, the patient's loss of consciousness is confirmed, and 0.6-0.9 mg / kg of muscle relaxant rocuronium is injected. The dial is then adjusted to provide an oxygen flow rate of 10 L / min and an inspiration desflurane concentration of 1 MAC. Endotracheal intubation is performed 90 seconds after muscle relaxant injection. Mechanical ventilation is then started and the oxygen flow rate is adjusted to 2 L / min. After endotracheal intubation, the dose of desflurane is adjusted according to the randomized group.

In the BIS-guided group, the dose of desflurane is adjusted so that the patient's BIS value is 50 (range 40-60). Desflurane’s end-tidal concentration is adjusted to 0.3-2.0 MAC. Target concentration control of remifentanil targets the systolic blood pressure of 120 (range 100-140) mmHg of the patient. When systolic blood pressure is 121-140mmHg, remifentanil is increased by 1ng / ml based on the effect site concentration, and when the systolic blood pressure is > 140mmHg, remifentanil is increased by 2ng / ml. If the systolic blood pressure is 100-120mmHg, remifentanil is reduced by 1 ng / ml based on the target concentration, and when <100 mmHg, 2 ng / ml is reduced.

In the fixed-dose group, the dose of inhalation anesthetics is adjusted to 1 MAC, age-adjusted, according to Mapleson's method (6.6 vol% for inspiratory desflurane 1 MAC at age 40). Target concentration control of remifentanil targets the systolic blood pressure 120 (range 100-140) mmHg of the patient. Increasing the BIS value above 60 increases the concentration of remifentanil by 1 ng / ml.

In both groups, 2 mg of midazolam administered and the study should be discontinued if the BIS value continues to be above 60 for more than 5 minutes even after sufficient remifentanil and inhalation anesthetics were administered according to the protocol. In both groups, the range of effective concentration of remifentanil is 1-20 ng / ml. In both groups, if the patient's systolic blood pressure is less than 100 mmHg and this continues for 10 minutes or more, even when the concentration of remifentanil infusion was sufficiently reduced to 1 ng / ml, proper administration of vasopressors should be given and the study should be discontinued. In both groups, if the patient's heart rate is maintained at less than 40 beats per minute for more than 10 minutes, atropine 0.5 mg is given and the study is discontinued.

In both groups, muscle relaxation during surgery is maintained using rocuronium. The total amount of muscle relaxant used is recorded after the end of surgery. Data for comparison between the two groups is collected as follows. During the operation, heart rate is continuously monitored and systolic blood pressure is measured every 2.5 minutes for noninvasive blood pressure. Changes in heart rate and systolic blood pressure (1 second ineterval data) during the operation are collected and recorded through the patient monitor 10 minutes after the start of the operation and 10 minutes before the end of the operation. The BIS value is recorded by copying the value stored in the USB memory to the computer at the interval of 1 second through the USB terminal of the BIS monitor after the operation.

In both groups, 5-6 ml / kg of crystalloid solution is administered during anesthesia induction, and 1.5-2 ml / kg of crystalloid solution is administered during anesthesia maintenance. If the hourly urine flow rate is less than 1 ml / kg, an additional 6 ml / kg of crystalloid fluid is administered.

Remifentanil continuous infusion is stopped when skin closure begins. At the end of skin closure, the concentration of inhalation anesthetics is adjusted so that the patient's end-tidal inhalation anesthetic concentration is 0.3MAC. At the end of the skin closure, the fresh gas flow rate is increased to 10 L / min, the reversal agent is administered and time measurement is started. At this time, the ventilation amount is controlled by one breathing volume of 8ml / kg and the respiratory rate of 12 breaths / minute. When the spontaneous breathing of the patient collides with the ventilator, manual ventilation is initiated to recover from general anesthesia. The time until the patient opens his/her eyes at various stimulus (voice stimulation, light tapping) is recorded.

On postoperative day 1, the patient is visited and a modified Brice questionnaire is used to check for arousal during surgery.

1. **Comparative group setting and random assignment method**

BIS-guided group: desflurane concentration is adjusted to a BIS value of 50 and the remifentanil concentration is adjusted to a systolic blood pressure of 120 mmHg during surgery.

Fixed-dose group: the inspiratory concentration of desflurane is controlled to 1 MAC and the concentration of remifentanil targets the systolic blood pressure of 120 mmHg.

Patients who meet the selection criteria will be included in the study if they agree in writing. Doctors who are not involved in the treatment assigns patients to each group in a randomly generated sequence by computer generated randomization. Induction and maintenance of general anesthesia are performed in a manner appropriate to the group in the randomization table.

1. **Reasons for selection and administration of test drug, method of administration and use, combination therapy and reference drug**

Not applicable

1. **Observation items, clinical test items and observation test methods**

The patient's heart rate, systolic blood pressure data, and BIS values from 10 minutes after surgery to 10 minutes before surgery are collected and compared at 1 second intervals.

After the discontinuation of the anesthetic agent, the time it takes for the patient to open his eyes for stimulation (voice stimulation, light tapping) is compared between the two groups.

The number of times to increase or decrease the concentration of Remifentanil and desflurane

Observe awakening during surgery.

1. **Evaluation criteria and evaluation methods**

The primary efficacy endpoint in this study was the performance measurement of BIS and systolic blood pressure during surgery in each group. The method of calculating PM values is described in the Data Analysis part.

The secondary efficacy endpoint in this study is the time from the discontinuation of anesthesia to the awakening of the patient. Awakening time is measured from the time of discontinuation of anesthesia to the patient's response to mild arousal (light tapping, negative).

1. **Differentiation from existing treatments and research**

Among previous studies on balanced anesthesia using inhalation anesthetics and opiate analogs, there has not been any studies on comparing the maintenance of the stability of anesthesia by controlling the concentration of inhalation anesthetics and maintaining a constant end-tidal concentration of inhalation anesthetics.

1. **Benefits and risks of research subjects**

The subjects of this study were patients who were scheduled for laparoscopic gastrectomy under general anesthesia. Currently, balanced anesthesia using desflurane and remifentanil together is a widely used method of general anesthesia and does not require additional treatment for the patient. In the present method, balanced anesthesia with BIS monitoring during general anesthesia can reduce the risk of arousal during surgery, and by reducing the amount of inhalation anesthetics, the hemodynamic change can be stably maintained and the time required for arousal can be reduced. Therefore, the patient's participation in this study has additional benefits compared to general anesthesia using only general inhalation anesthesia, but there is no risk to the patient.

1. **Dropout Criteria**

Subject's Withdrawal of Consent

If the investigator believes that the progress of the study is in violation of the patient’s needs

Patients who need transfusion during general anesthesia or who have received one or more units of red blood cells

Patients with BIS> 60 continuously for more than 5 minutes

Heart rate less than 40bpm for more than 10 minuties under general anesthesia

Systolic blood pressure less than 100 mmHg for more than 10 minutes (requiring continuous infusion of vasopressors)

1. **Evaluation criteria, evaluation methods and reporting methods of safety including side effects**

All patients participating in this study should be subjected to a safety assessment.

The patient's vital signs and BIS are monitored during anesthesia and the patient's vital signs are continuously monitored until he or she exits to an anesthetic recovery or intensive care unit. Recording of adverse reactions occur during the study and any causal relationship with the methods used in the study are evaluated. The severity, duration, and causal relationship are analyzed. The treatment and results for adverse events are recorded. Adverse events are assessed by the investigator by monitoring the patient's condition continuously during anesthesia, from the end of the anesthesia to the exit of the recovery or intensive care unit. Clinical data are analyzed using appropriate statistical methods such as intra- and post-treatment comparisons according to the characteristics of the variables, and the frequency of adverse reactions, expression rates, lists, severity, and causal relationship with test methods are presented. Report as a graph if necessary. Adverse events should be reported to the Medical Research Ethics Review Board on the following principles:

1. Scope of Reporting Suspected Unexpected Serious Adverse Reaction

1) When all of the following ①-③ are satisfied as an adverse reaction occurred in domestic and overseas clinical trials conducted under the same protocol as the clinical trial approved by the institution

① Unexpectedness

(A) not observed in previous or previous trials;

(B) does not match the approved product information;

② Relatedness

③ Seriousness

(A) death

(B) life-threatening

(C) If it causes hospitalization or an extension of hospitalization period.

(D) results in sustained or significant impairment or loss of function;

(E) causing congenital malformations or abnormalities;

(F) important medical events

(G) Other

2) Among other unforeseen adverse events, the investigator may change the IRB's assessment of the risks and potential benefits of the study, regardless of severity, and as a result, change the plan or consent process is considered.

3) Others include reporting of adverse reactions other than those specified in the protocol or the investigator's data sheet.

2. Deadline for Reporting Suspected Unexpected Serious Adverse Reactions

1) The lead investigator shall promptly report all adverse reactions in the case of critical, unforeseen, and related studies, procedures, or drug / medical device / biological products within the timeframes set out below.

① In case of death or life threatening, the researcher shall report within 7 days of receiving or knowing this fact, and report detailed information within 8 days of the initial report date. If the death was reported, additional information such as an autopsy report (if an autopsy was performed) and a death certificate may be provided.

② All other serious and unexpected adverse drug / medical reaction reports must be reported within 15 days of the date the investigator received or learned of this fact.

③ If there is additional information on the reported adverse drug substance / device reaction, it should be reported until the end of the drug / medical device reaction (that is, the abnormal drug / medical device reaction disappears or becomes impossible to follow up). .

3. Scope of reporting safety related information

1) Major adverse reactions other than those subject to SUSARs that occurred in the same study protocol

2) Report of adverse drug / medical device reactions occurring in other institutions and overseas clinical trials conducted under the same protocol as the clinical trial product / medical device, but with a different protocol than that approved by this institution.

3) reporting safety-related issues that may have a significant impact on the safety of the study subjects and the conduct of the study or which may change IRB decisions;

4) Report of changes in the clinical investigator data sheet and report of decisions made by the Data Safety Monitoring Committee

5) Quarterly safety related information report submitted from the sponsor

6) Abnormal drug / medical device reaction reports reported from drugs already on the market

4. Deadline for reporting safety information

1) The lead investigator shall report all information related to adverse reactions and other safety outside the scope of adverse drug / medical device reaction report through the safety related information report.

① A summary of accumulated data, not individual cases, shall be reported at regular reporting cycles. (E.g., if the interim reporting period is 3 months, collect and report safety related information for 3 months and report it.)

② If there is any additional information, the report should be reported until the end of the adverse drug reaction (that is, the abnormal drug reaction disappears or becomes impossible to follow up).

5. Definition of severity and causality.

1) Severity

Adverse events are classified into mild, moderate and severe according to the definition below.

Mild: does not interfere with normal, temporary, or routine activities.

Moderate: illness may cause some discomfort or interfere with daily activities.

Severe: cannot carry out daily activities.

2) causality

 Causality can be divided into 'not thought to be related', 'possibly related', 'likely', 'obviously related', or 'difficult to identify relevance'.

**10) Data Safety Monitoring Plan (DSMP)**

In order to protect the rights and welfare of the subjects, all the subjects' data should be prepared in case report papers or encrypted electronic files and stored in the designated repository. In accordance with the GCP guidelines, this study will ensure that supervisors who are unrelated to the study verify the accuracy, completeness and verifiability of the information recorded in the case record. All patients participating in the study will participate in the study after writing informed consent.

**11) Data analysis and statistical analysis method**

In this study, we will perform the performance measurement (PM) method to compare systolic blood pressure and BIS data between two groups. PM was originally designed to post-evaluate the mechanical suitability of intravenous pumps for total intravenous anesthesia by target concentration injection, or the appropriateness of the pharmacokinetic model or infusion control algorithm of drugs used in the pump (Ref. 3). However, this method may be used to assess the patient's response to a specific treatment by expanding its application (Ref. 4). The PM is calculated by first calculating the performance error (PE) from the target and actual measurements of the treatment, from 1) median performance error (MDPE), 2) median absolute performance error (MDAPE), 3) wobble, and 4) divergence. These values represent the difference in the target value and actual values: 1) bias 2) accuracy 3) stability 4) improvement over time.

An example of PM calculation in systolic blood pressure is as follows.

PE*ij* = $\frac{\mathrm{SAPm}ij-\mathrm{SAPt}ij}{\mathrm{SAPt}ij}$ x 100

MDPE*i* = median{PE*ij*, *j*=1,…,N*i*}

MDAPE*i* = median{|PE*ij*|, *j*=1,…,N*i*}

Wobble*i* = median{|PE*ij*-MDPE*i*|, *j*=1,…,N*i*}

Divergence = slope{|PE*ij*|, *j*=1,…,N*i*}

SAP=systolic arterial pressure; m=measured; t=target 100; MDPE=median PE, MDAPE=median absolute PE, slope=slope of the regression curve of |PE|’s

This study takes a conservative approach to the analysis of all patients (48) who participated in the ITT protocol. The statistical program SPSS 21.0 and Microsoft Excel 2010 are used. Both average and fractional comparisons between groups are performed using nonparametric methods. P <0.05 is considered statistically significant.

**12) 연구수행일정표**

12 months from IRB approval date

Assuming that data can be collected from surgery under an average of 20 general anesthesia each week, approximately six months are required for 48 data collections. A six month time period for supplemental studies and statistical treatment is estimated to estimate the study period of 12 months after IRB approval.

1. **Measures for the Safety Protection of Research Subjects**
2. **Basic plan for securing research ethics**

In accordance with the 2013 Helsinki Declaration, a written agreement will be obtained from the subject (or guardian) after the subject or guardian has been fully informed of the purpose of the study and the possible psychological and physical hazards involved in the study. Only the subject, the subject's physician, and the participant will be kept informed of the subject's participation in the test or the progress of the treatment, and the records that identify the subject's identity will be kept confidential. Unnecessary personal identifiers of the data collected will be eliminated. In particular, case records will not include the patient's name, social security number, chart number, etc., and the identifier code associated with the personal information will be managed separately. When submitting a photograph related to a patient, the patient's identity will not be known, and if there is a possibility of any identity being disclosed, it will state that written consent has been obtained. Subject screening records will be kept confidential and will not be transferred elsewhere and may be sent to a supervisory authority to be supervised by the progress of the study. Subject screening records will be kept confidential and will not be transferred elsewhere and may be sent to a supervisory authority to be supervised by the progress of the study. Data collected for the study is kept for three years after the study ends. Documents beyond the retention period will be destroyed in accordance with Article 16 of the Enforcement Decree of the Personal Information Protection Act. Store in accordance with the Bioethics and Safety Act. This study will comply with the Ethics Regulations of the Hospital Ethics Committee and the ICH-GCP.

1. **Consent Process**

The investigator explains to the patient according to the attached explanations and informed consent and obtains written consent. Consent must be limited to the subjects, their spouses and children. Allow sufficient time for the patient to voluntarily agree after the study description. There will be no coercion in the consent process and the explanation will be conducted in plain language that is acceptable to the public. After obtaining consent, the patient should keep a copy of the explanation.

1. **Compensation Plan for Research Subjects**

Not applicable

1. **Measures to protect personal information of research subjects**

Records identifying the subject's identity will be kept confidential. Unnecessary personal identifiers will be removed from the data collected, and in particular, case records will not include the patient's name, social security number, or chart number. In the case of electronic files, encrypt them and keep them in the designated storage. When submitting a photograph related to a patient, the patient's identity will not be known, and if there is a possibility of any identity being disclosed, it will state that written consent has been obtained. Subject screening records will be kept confidential and will not be transferred elsewhere and may be sent to a supervisory authority to be supervised by the progress of the study. Subject screening records will be kept confidential and will not be transferred elsewhere and may be sent to a supervisory authority to be supervised by the progress of the study. Data collected for the study is kept for three years after the study ends.

1. **Additional protection measures when including vulnerable subjects**

Not applicable

1. **References**
2. Manyam SC, Gupta DK, Johnson KB, White JL, Pace NL, Westenskow DR, et al. Opioid–Volatile Anesthetic Synergy: A Response Surface Model with Remifentanil and Sevoflurane as Prototypes. Anesthesiology. 2006;105(2):267-78.
3. Avidan MS, Zhang L, Burnside BA, Finkel KJ, Searleman AC, Selvidge JA, Saager L, Turner MS, Rao S, Bottros M, Hantler C, Jacobsohn E, Evers AS. Anesthesia awareness and the bispectral index. N Engl J Med. 2008 Mar 13;358(11):1097-108.
4. Varvel JR, Donoho DL, Shafer SL. Measuring the predictive performance of computer-controlled infusion pumps. J Pharmacokinet Biopharm. 1992;20(1):63-94.
5. Struys MM, De Smet T, Versichelen LF, Van De Velde S, Van den Broecke R, Mortier EP. Comparison of closed-loop controlled administration of propofol using Bispectral Index as the controlled variable versus "standard practice" controlled administration. Anesthesiology. 2001;95(1):6-17.
6. Pollard RJ, Coyle JP, Gilbert RL, Beck JE. Intraoperative awareness in a regional medical system: a review of 3 years' data. Anesthesiology. 2007;106(2):269-74.
