# Supplementary material for: Comparison of bispectral index-guided and fixed-gas concentration techniques in desflurane and remifentanil anesthesia: A randomized controlled trial
Source: PLoS One. 2020 Nov 5;15(11):e0241828. doi: 10.1371/journal.pone.0241828 (PMC7644031; doi:10.1371/journal.pone.0241828)
Supplement: S1 Material — (ZIP) [file pone.0241828.s004.zip › Study protocol Original Korean.docx]

**Desflurane-Remifentanil 균형 마취에서 Desflurane 투여 방법 비교: Bispectral Index에 의한 조절법 대 고정 농도 주입법**

**Bispectral Index-Guided versus Fixed Dose Administration of Desflurane during Balanced Anesthesia with Remifentanil**

**Version No: 1.2**

**책임연구자 소속: 마취통증의학과**

**책임연구자 이름: 정철우**

**연구 개요**

| 연구제목 | (국문) Desflurane-Remifentanil 균형 마취에서 Desflurane 투여 방법 비교: Bispectral Index에 의한 조절법 대 고정 농도 주입법 |
| --- | --- |
|  | (영문) Bispectral Index-Guided versus Fixed Dose Administration of Desflurane during Balanced Anesthesia with Remifentanil |
| 책임연구자 | 정철우 |
| 연구비 지원기관 | 서울대학교병원 |

| 연구 목적 | 흡입마취제 desflurane과 아편유사제 remifentanil을 이용한 균형 마취시 desflurane 투여량을 Bispectral Index (BIS) 값 50을 목표로 조절한 경우와 desflurane 1 최소폐포농도(Minimum Alveolar Concentration, MAC)로 고정 투여하는 경우의 임상적 차이를 비교한다. |
| --- | --- |
| 연구 설계 | 전향적 무작위 임상시험 |
| 연구 기간 | IRB승인일로부터 12개월 |
| 연구 대상 | 전신 마취 하 복강경 위 절제술을 받는 환자 |
| 연구 대상자 수 | 48명 |
| 취약한 연구대상자 | 취약한 연구 대상자를 포함하지 않음 |
| 연구 방법 | Desflurane과 remifentanil을 이용한 균형 마취를 시행한다. 무작위로 배정된 군에 따라 한 군은 BIS 값 50 (범위 40-60)을 유지하는 것을 목표로 desflurane의 호기말 농도를 조절하고, 다른 군은 desflurane의 투여 농도를 1 MAC으로 고정하여 유지한다. 이 때 remifentanil의 투여는 desflurane과 별개로 두 군 모두 수축기 혈압을 120mmHg (범위 100-140mmHg)로 유지하는 것을 목표로 조절한다. |
| 유효성 평가 | 마취 유지 중의 두 군간의 BIS 값과 생체 징후의 안정성을 비교 평가한다. 수술 종료로부터 각성하기까지의 시간을 비교한다. |
| 안전성 평가 | 본 시험에 참여한 모든 환자를 대상으로 BIS 값 및 생체 징후의 이상값을 관찰하고 평가한다. 수술 중 각성의 발생 여부를 평가한다. |
| 기대효과 및  예상결과 | 균형마취의 방법 중, 이론적으로 우수한 방법인 BIS 값에 따라 desflurane을 조절하여 투여하는 경우보다 임상적으로 편의성이 높은 방법인 흡입 마취제의 농도를 1 MAC으로 고정하여 투여한 군에서 마취 유지 중 수면 상태와 생체 징후가 더욱 안정적으로 유지될 것이다. |

**연구계획서**

1. **연구 제목**

Desflurane-Remifentanil 균형 마취에서 Desflurane 투여 방법 비교: Bispectral Index에 의한 조절법 대 고정농도 주입법

1. **연구의 실시기관 명칭 및 주소**

서울대학교병원 마취통증의학과, 서울특별시 종로구 연건동 28

1. **연구책임자 및 공동연구자 성명 및 직명**
2. **연구책임자**

정철우

부교수

서울특별시 종로구 연건동 28, 서울대학교병원 마취통증의학과

전화번호: 02-2072-0640

1. **공동연구자**

해당없음

1. **연구담당자**

정유선

전임의

서울특별시 종로구 연건동 28, 서울대학교병원 마취통증의학과

전화번호: 02-2072-7361

1. **임상시험용 의약품 관리약사 / 임상시험용 의료기기 관리자**

해당없음

1. **연구 의뢰기관
   1) 연구 의뢰기관 명칭 및 주소**

없음

**2) 모니터요원 성명 및 직명**

이용헌

진료교수

서울특별시 종로구 연건동 28번지, 서울대학교병원 마취통증의학과

Tel. 02-2072-0779

1. **연구비 지원기관 명칭 및 주소**

없음

1. **예상연구기간**

IRB 승인일로부터 12개월

1. **연구 대상**

전신 마취하 복강경 위 절제술을 받는 환자

1. **연구의 배경 및 목적**

**1) 연구 배경**

현대 마취에서의 ‘균형 마취(balanced anesthesia)’란 흡입 마취제에 적정량의 아편 유사제를 병용하는 전신 마취의 방법을 의미한다. 이 방법은 적절한 양의 아편 유사제를 병용함으로써 흡입 마취제만을 사용하는 경우에 비해 흡입 마취제의 과량 사용에 따른 부작용, 즉 혈역학적 불안정, 각성 시간의 지연 등을 줄일 수 있는 이점이 있다. 그러나 아편 유사제를 과량으로 투여하거나 흡입 마취제 농도를 지나치게 낮추는 경우에는 오히려 저혈압, 서맥 또는 수술중 각성 등의 문제가 발생할 수 있다. 균형 마취를 시행함에 있어 기대하는 효과를 높이고 부작용을 줄이기 위해서는 아편 유사제와 흡입 마취제의 투여량 사이의 균형을 유지하는 것이 중요하다.

균형 마취에서의 ‘균형’은 이론적으로는 흡입 마취제와 아편 유사제의 동일 효과 곡선(isobologram) 상의 어떤 조합을 통해서도 가능하다. Manyam 등에 따르면, 흡입마취제를 최소화하고 이를 보조하는 적절한 양의 아편 유사제를 병용하는 것이 권장되는데, 이 경우 안정적인 혈역학적 상태와 함께 가장 빠른 각성 시간을 보장할 수 있기 때문이다. Sevoflurane과 remifentanil의 균형 마취시 이에 해당되는 조합은 sevoflurane 0.35-0.5 MAC과 remifentanil 효과처 농도 5-7 ng/ml 정도이다. 그러나 임상에서 이처럼 낮은 농도의 흡입 마취제를 사용할 경우 수술중 자극의 변화에 따라 수술중 각성이 발생할 가능성이 있으므로, 흡입 마취제의 사용을 최소화하기 위해서는 환자의 각성도를 BIS 등을 이용하여 감시하며 BIS 40-60을 목표로 흡입 마취제를 조절하여 사용하는 것이 필요하다. 이 때 remifentanil은 흡입 마취제와는 별도로 혈역학적 상태에 따라 조절이 필요하다.

그러나 이러한 이론적인 방법을 임상에 그대로 적용하기에는 여전히 많은 수술장이 BIS를 이용한 감시가 불가능한 현실이 큰 장애가 된다. 좀 더 보편적이고 실용적인 방법은 각성의 가능성이 없는 최소한의 농도 이상으로 흡입마취제 투여량을 고정 투여하는 방법이다. Avidan 등은 흡입 마취제에서 BIS 감시 없이도 수술중 각성의 문제가 안 생길 최소한의 농도는 약 0.7-1.3 MAC으로 추정하였으며 평균 호기말농도 약 0.8 MAC에서 BIS 40-60이 안정적으로 유지되는 것을 보고하였다. 따라서 BIS 감시 없이도 실행 가능한 안정적인 실용적인 균형 마취의 방법은 호기말 농도 0.8 MAC의 흡입마취제를 고정적으로 투여하고 혈역학적 상태에 따라 병용하는 remifentanil의 투여량을 조절하는 방법일 것이다.

**2) 연구 가설 및 목적**

본 연구에서는 균형 마취에서 흡입 마취제를 조절하는 두 가지 방법을 비교하고자 한다. BIS 값 40-60을 목표로 흡입 마취제를 조절하여 사용하는 방법(BIS-guided technique)은 이론적으로 우수한 배경을 갖고 있다. 이 방법은 흡입 마취제 사용량을 매우 줄일 수 있어 수술 후 각성 속도의 측면에서 이점이 있으며 충분한 아편 유사제의 사용으로 혈역학적 안정을 얻을 수 있는 이점이 있는 반면, 흡입 마취제 및 아편 유사제의 투여량을 수시로 조절해야 해서 오히려 안정적인 마취가 이루어지지 못하거나 또는 마취의의 심각한 피로를 유발할 수 있다.

흡입 마취제를 투여량 1 MAC(이 경우 호기말 농도가 0.8 MAC 정도가 된다)에 고정하여 투여하고 (Fixed dose technique) 아편 유사제를 이용하여 혈역학적 상태를 조절하는 방법은 임상에 적용하기 쉬운 실용적인 방법이다. 상대적으로 높은 농도의 흡입 마취제를 사용하는 데 따른 저혈압 및 수술 후 각성의 지연 등이 발생할 가능성이 있는 점이 단점으로 지적될 수 있으나, 잦은 조작의 필요가 적어 마취의의 수고를 덜 수 있고 앞의 방법에 비해 더 안정적인 BIS 값과 생체 징후의 유지가 가능한 장점이 있을 것이다. 본 연구에서는 이 두 방법을 임상적인 효용성과 안정성 측면에서 비교 분석하여 실용적인 균형마취 방법(fixed dose technique)의 이점을 제시하고자 한다.

1. **임상시험용 의약품 및 의료기기 코드명(또는 주성분의 일반명), 원료약품의 분량, 제형 등(대조약 포함)**

해당사항 없음

1. **연구대상자의 선정 기준, 제외기준, 목표한 대상자 수 및 산출 근거**
2. **선정기준**

정규 수술로 전신 마취 하 복강경 위 절제술을 받는 환자 중 흡입 마취제 desflurane과 아편 유사제 remifentanil을 이용한 균형 마취의 적응증이 되는 환자

병동에서 측정한 수축기 혈압의 평균이 100-140mmHg인 환자

1. **제외기준**

시험에 동의하지 않는 환자

흡입 마취제 desflurane과 아편 유사제 remifentanil을 이용한 균형 마취 외 다른 마취 방법으로 전신마취를 시행하는 환자

신경근 차단제, 마취제, 아편 유사제에 알러지가 있는 환자

악성 고열증의 과거력이 있는 환자

중추신경 작용제를 복용중인 환자

만성 알코올 중독자

발작성 질환으로 치료중인 환자

중중의 심기능 이상 환자 (EF<30%)

미국 마취과학회 등급 IV 이상인 환자

마취 시작 전 수축기 혈압이 90mmHg 이하인 환자

수술 중 생체 징후가 혈역학적으로 불안정하여 승압제 지속 주입을 필요로 하는 환자

마취 시간이 30분 이내인 환자

만성 고혈압 환자

허혈성 심질환 환자

뇌 허혈성 질환이 있는 환자

베타 차단제 혹은 항부정맥제를 복용중인 환자

1. **목표한 대상자 수 및 산출 근거**

IRB 승인 후 각 군당 21명씩 명의 환자를 대상으로 결과를 수집하여 분석한다. 탈락률 10%를 감안하여 각 군당 24명씩을 모집할 계획이다.

[산출근거]

본 연구에서의 일차 목표는 두 군 사이의 ‘마취의 안정성(stability)’의 차이를 BIS, 수축기 혈압 각각에서 비교하여 fixed dose technique의 우수성을 보이는 것이다. 그러나 균형 마취의 본질적인 특징으로 인해 수축기 혈압의 차이는 적을 것으로 생각되고, 두 군의 주된 차이는 BIS값의 안정성의 차이로 나타날 것으로 기대된다. BIS의 안정성은 PM (아래 11번 통계 방법에 기술)의 변수 중 wobble (값이 낮을수록 안정적)로 표현되는데, 기존에 균형마취를 받은 환자들 중 본 연구의 두 군인 BIS-guided group과 fixed dose group의 프로토콜과 유사하게 마취가 진행된 환자 각 4명, 3명의 저장된 BIS 기록(1분 간격 데이터가 장치 내부의 메모리에 자동 저장됨)으로부터 BIS의 PM을 계산하였을 때 MDPE는 각각 -3.2+/- 5.6%, -24+/- 11.2%였으며 wobble은 각각 평균 10%, 6% (표준편차 5%)로 계측되었다. 이에 따라 각 군에서 필요한 대상수를 One-tailed, 제 1종 오류(α) 0.05, power 0.8을 가정하여 계산하였을 때, 각 군은 wobble의 평가시 최대 21명씩 필요하며 탈락률 10%를 가정하였을 때 각 군 24명씩 총 48명이 필요할 것으로 예상된다.

1. **연구 대상자 모집 계획**

전신 마취하에 수술을 받기로 예정된 20세에서 80세 사이의 환자들에게 별첨된 설명문과 동의서에 따라 연구 담당자가 환자에게 설명한 후 서면 동의를 받는다. 본 연구의 책임연구자 및 의료기관은 인종이나 사회 경제적 상태에만 근거해서 이 연구에 참여할 가능성이 있는 환자를 배제시키지 않을 것이다.

1. **연구 방법**
2. **구체적인 연구방법**

전신 마취 하에 복강경 위 절제술을 받는 환자들을 대상으로 연구를 시행한다. 마취 유도 전 환자들을 무작위로 배정된 순서에 따라 BIS-guided group 24명, Fixed-dose group 24명으로 나눈다.

마취 유도의 방법은 두 군에서 동일하게 시행한다. 환자가 수술실에 도착하면 심전도와 맥박산소계측기, 비침습적 혈압감시장치, BIS 모니터를 부착하고 100% 산소로 전산소화한다. 전신마취의 유도는 propofol과 아편유사제인 remifentanil을 이용한다. 환자의 상태가 안정된 후 레미펜타닐을 Minto model에 의한 목표농도조절주입법을 이용하여 효과처 농도 5ng/ml를 목표로 주입한다. 목표 농도에 도달하면 1% propofol 1.2 mg/kg를 주입한다. 30초 후 환자의 의식 소실을 확인하고 근이완제 rocuronium을 0.6-0.9 mg/kg 주입한다. 이어서 산소 유량 10 L/min, 흡입 desflurane 농도 1 MAC이 되도록 다이얼을 조정하고 용수환기를 시행한다. 근이완제 주입 시점으로부터 90초 후 기관내 삽관을 시행한다. 이후 기계 환기를 시작하고, 산소 유량은 2 L/min으로 조절한다. 기관내 삽관이 이루어진 후 무작위 배정된 군에 따라서 desflurane의 투여량을 조절한다.

BIS-guided group의 경우 환자의 BIS 값이 50 (범위 40-60)이 되도록 desflurane의 투여량을 조절한다. Desflurane의 경우 호기말 농도가 0.3-2.0MAC이 되도록 조절한다. Remifentanil의 목표 농도 조절은 환자의 수축기 혈압 120 (범위 100-140) mmHg를 목표로 조절한다. 수축기 혈압이 121-140mmHg인 경우 remifentanil을 효과처 농도 기준 1ng/ml 증량하고, >140mmHg인 경우 2ng/ml 증량한다. 수축기 혈압이 100-120mmHg인 경우 remifentanil을 효과처 농도 기준 1ng/ml 감량하고, <100mmHg인 경우 2ng/ml 감량한다.

Fixed-dose group의 경우 흡입마취제의 투여 농도가 Mapleson의 방법에 따라 나이에 맞게 보정된 1 MAC이 되도록 조절한다 (40세를 기준으로 inspiratory desflurane 1 MAC은 desflurane 6.6 vol%). Remifentanil의 목표 농도 조절은 환자의 수축기 혈압 120 (범위 100-140) mmHg를 목표로 조절한다. BIS 값이 60 이상으로 증가하는 경우 레미펜타닐의 효과처 농도를 1ng/ml 증가시킨다.

두 군 모두에서 프로토콜에 따라 레미펜타닐과 흡입 마취제를 충분히 투여하여도 BIS 값이 60 이상으로 5분 이상 높게 유지되는 경우 midazolam 2mg을 투여하고 연구 진행을 중지한다. 두 군 모두에서 레미펜타닐의 효과처 농도 범위는 1-20ng/ml로 한다. 두 군 모두에서 레미펜타닐 지속 주입 효과처 농도가 1ng/ml로 충분히 감소시킨 상태에서도 환자의 수축기 혈압이 100mmHg 미만으로 10분 이상 지속시 지속적 승압제 주입을 시행하고 연구 진행을 중단한다. 두 군 모두에서 환자의 심박수가 분당 40회 미만으로 10분 이상 유지되는 경우 아트로핀 0.5mg을 정주하고 연구 진행을 중단한다.

두 군 모두 수술 중 근이완은 rocuronium을 이용하여 유지한다. 수술 종료 후 근이완제 사용량을 기록한다. 두 군 간의 비교를 위한 데이터는 다음과 같이 수집한다. 수술 중 심박수는 연속적으로 감시하고 수축기 혈압은 2.5분에 한 번씩 비침습적 혈압을 측정한다. 수술 중의 심박수 및 수축기 혈압의 변화는 환자 모니터의 디지털 아웃단자를 통해 수술 시작 10분 후부터 수술 종료 10분 전까지의 1초 단위 데이터를 수집하여 기록한다. BIS 값은 BIS 모니터의 USB 단자를 통해 1초 간격으로 USB 메모리에 저장된 값을 수술 종료 후 컴퓨터로 복사하여 기록한다.

두 군 모두 마취 유도시 5~6ml/kg의 정질액을 투여하게 되며, 마취 유지시에는 1.5~2ml/kg의 정질액을 투여한다. 시간당 소변 유량이 1ml/kg 미만일 경우에는 6ml/kg의 정질액을 추가로 투여한다.

피부 봉합이 시작되면 레미펜타닐 지속 주입을 중단한다. 피부 봉합이 끝나는 시점에 환자의 호기말 흡입 마취제 농도가 0.3MAC이 되도록 흡입 마취제의 농도를 조절한다. 피부 봉합이 끝나면 신선 가스 유량을 10L/분으로 증가시키고 근이완 역전제를 투여하고 시간 측정을 시작한다. 이때 환기량은 일회 호흡량 8ml/kg, 호흡수는 12회/분으로 조절하며 환자의 자발 호흡이 마취기와 충돌하면 용수 환기로 전환하여 전신 마취 회복을 시행한다. 환자가 자극(음성 자극, 가벼운 두드림)에 대해 눈을 뜨기까지의 시간을 기록한다.

수술 다음 날 환자를 방문하여 modified Brice questionnaire [별첨2] 를 이용하여 수술중 각성의 발생 여부에 대한 확인을 한다.

1. **비교군 설정 및 무작위 배정 방법**

BIS-guided group: 수술 중 desflurane의 농도를 BIS 값 50을 목표로 조절하고, remifentanil의 농도를 수축기 혈압 120mmHg를 목표로 하여 조절하는 군

Fixed-dose group: 수술 중 desflurane의 흡기 농도를 1 MAC으로 고정하고 remifentanil의 농도를 수축기 혈압 120mmHg를 목표로 하여 조절하는 군

선정 기준에 적합한 환자가 연구 시행에 서면으로 동의하면 연구 대상에 포함시킨다. 진료에 관여하지 않은 의사가 computer generated randomization으로 무작위로 생성된 순서에 따라 환자를 각 군에 배정한다. 이후 무작위 배정표에 해당하는 군에 맞는 방법으로 전신 마취의 유도 및 유지를 시행한다.

1. **시험약 투여∙사용량, 투여∙사용 방법, 병용 요법, 대조약 사용시 그 선택사유**

해당사항 없음

1. **관찰항목, 임상검사항목 및 관찰검사방법**

수술 시작 10분 후부터 수술 종료 10분 전까지의 환자의 심박수와 수축기 혈압 데이터, BIS 값을 1초 간격으로 수집하여 비교한다.

마취 약제 중단 후 환자가 자극(음성 자극, 가벼운 두드림)에 대해서 눈을 뜨기까지 소요된 시간을 두 군간에 비교한다.

Remifentanil과 desflurane의 농도를 증감량하는 횟수

수술 중 각성의 발생 여부를 관찰한다.

1. **효과 평가기준, 평가 방법**

본 연구의 일차 유효성 평가변수는 각 군에서의 수술 중 BIS, 수축기 혈압의 performance measurement 값이다. PM값의 계산 방법은 11 자료 분석에 설명되어 있다.

본 연구의 이차 유효성 평가변수는 마취제 투여 중단 후 환자가 각성하기까지의 시간이다. 각성 시간은 마취제 투여 중단시부터 환자가 가벼운 각성자극 (가벼운 두드림, 음성자극)에 반응하기까지의 시간을 측정한다.

1. **기존 치료 및 연구와의 차별점**

흡입 마취제와 아편 유사제를 병용하는 균형 마취에 대한 기존의 연구 중, BIS를 목표로 흡입마취제의 농도를 조절하는 방법과 흡입마취제의 호기말 농도를 일정하게 유지하는 방법을 마취 유지의 안정성(stability) 측면에서 비교한 연구는 없었다.

1. **연구대상자의 이익과 위험**

본 임상시험의 대상이 되는 환자군은 전신마취 하 복강경 위 절제술이 예정된 환자들이다. 현재 desflurane과 remifentanil을 함께 사용하는 균형 마취 자체는 전신 마취의 방법으로 이미 널리 사용되는 방법으로서 본 연구를 위해 환자에게 추가적인 처치가 필요하지는 않다. 본 연구의 방법에서 전신 마취 중 BIS 감시를 하면서 균형 마취를 실시함으로써 수술 중 각성의 위험을 줄일 수 있고 흡입 마취제 사용량을 줄임으로써 혈역학적 변화를 안정적으로 유지하고 각성에 소요되는 시간도 줄일 수 있다. 따라서 환자가 본 연구에 참가함으로써 일반적인 흡입 마취제만을 이용한 전신 마취의 경우에 비해 추가적으로 얻는 이득은 있으나 환자가 부담하게 되는 위험은 없다고 할 수 있다.

1. **중지∙탈락 기준**

피험자의 동의 철회

연구자가 연구의 진행이 환자의 이익에 위배된다고 판단되는 경우

전신마취 중 임상적으로 수혈을 필요로 한다고 판단되는 환자, 혹은 1 unit 이상의 적혈구를 수혈받은 환자

지속적으로 5분 이상 BIS>60인 환자

전신마취 중 심박수가 40회 미만으로 10분 이상 지속되는 경우

전신마취 중 지속적 승압제 주입을 필요로 하는 경우 (수축기 혈압이 100mmHg 미만으로 10분 이상 지속되는 경우)

1. **부작용을 포함한 안전성의 평가기준, 평가 방법 및 보고 방법**

본 시험에 참여한 모든 환자를 대상으로 안전성 평가를 실시한다.

마취 중 지속적으로 환자의 생체 징후와 BIS를 감시하며, 마취 회복실 혹은 중환자실로 퇴실하기까지 지속적으로 환자의 생체 징후를 감시한다. 연구 시행 중에 이상반응이 발생했을 경우, 연구에 사용된 시험 방법과의 인과 관계 유무와 모든 이상 반응을 기록하고 추후 중증도, 중대성, 기간, 그리고 시험 방법과의 인과관계를 평가한다. 이상반응에 대한 처치 및 결과 역시 기록한다. 이상 반응은 마취 중, 마취 종료 후 회복실 혹은 중환자실 퇴실까지 지속적으로 연구자가 환자의 상태를 감시하여 평가하게 되며 필요한 경우 이후에도 추가적인 검사와 검진을 통해 평가하고 즉각적인 조치를 시행한다. 임상병리검사 자료에 대해서는 변수의 특성에 따라 치료 전, 후의 군내 비교 등 적절한 통계적 방법을 이용하여 분석하고, 이상반응의 빈도, 발현율, 각각의 목록, 심각한 정도 및 시험 방법과의 인과관계 등을 제시하며, 필요한 경우 그래프 형태로 보고한다. 이상 반응에 대해서는 의학연구윤리심의위원회에 다음과 같은 원칙으로 보고한다.

1. 이상 약물/의료기기 반응(Suspected Unexpected Serious Adverse Reaction) 보고 범위

1) 본 기관에서 승인된 임상시험과 동일한 계획서로 실시되는 국내 및 해외 임상시험에서 발생한 이상반응으로서 아래 ①-③ 모두를 만족하는 경우

① 예상하지 못하고(Unexpectedness)

(가) 이전의 임상시험 또는 전 임상시험에서 관찰되지 않은 사항

(나) 승인 받은 제품 정보와 일치하지 않은 사항

② 관련성이 있으며(Relatedness)

③ 중대한 이상반응(Seriousness)

(가) 사망(death)

(나) 생명을 위협하는 경우(life-threatening)

(다) 입원 또는 입원기간의 연장을 초래하는 경우

(라) 지속적인 또는 중대한 불구나 기능 저하를 초래하는 경우

(마) 선천성 기형 또는 이상을 초래하는 경우

(바) 중요한 의학적 사건

(사) 기타

2) 그 외 예상하지 못한 이상반응 중에서, 연구자가 심각성(severity)과 상관 없이, 연구의 위험과 잠재적 이익에 대한 IRB의 평가를 변경시킬 수 있으며 그 결과 계획서나 동의 과정의 변경이 고려된다고 판단한 경우

3) 그 외에 임상시험계획서나 임상시험자자료집에서 즉시 보고하지 않아도 된다고 정한 사항 외의 기타 이상반응 보고 등을 포함한다.

2. 이상 약물/의료기기 반응(Suspected Unexpected Serious Adverse Reaction) 보고 기한

1) 책임연구자는 중대하고, 예상하지 못하며, 연구설계, 시술, 또는 약물/의료기기/생물학적 제제 등에 관련된 경우의 모든 이상반응을 아래에서 정한 기간 내에 신속히 보고하여야 한다.

① 사망을 초래하거나 생명을 위협하는 경우에는 연구자가 이 사실을 보고받거나 알게 된 날로부터 7일 이내에 보고하고, 상세한 정보를 최초보고일로부터 8일 이내에 추가로 보고하여야 한다. 사망사례를 보고한 경우 부검보고서(부검을 실시한 경우)와 사망진단서 등의 추가적인 정보를 제공 할 수 있다.

② 다른 모든 중대하고 예상하지 못한 이상약물/의료기기반응보고의 경우에는 연구자가 이 사실을 보고받거나 알게 된 날로부터 15일 이내에 보고하여야 한다.

③ 보고한 이상약물/의료기기 반응의 추가적인 정보가 있는 경우에는 해당 이상약물/의료기기반응이 종결(이상약물/의료기기 반응이 사라지거나 추적조사가 불가능하게 되는 것을 말함)될 때까지 보고하여야 한다.

3. 안전성 관련 정보 보고 범위

1) 동일한 임상시험 계획서에서 발생한 이상약물/의료기기반응보고(SUSAR) 대상 이외의 중대한 이상반응 보고

2) 동일한 임상시험용 제제/의료기기이지만 본 기관에서 승인된 임상시험과 다른 계획서로 실시되는 타기관 및 해외 임상시험에서 발생한 이상약물/의료기기 반응보고 등

3) 연구대상자의 안전과 연구의 실시에 중대한 영향을 줄 수 있거나 IRB 결정사항을 변경할 만한 안전성 관련 사항 보고

4) 임상시험자자료집 변경 보고, 자료안전성모니터링위원회 결정 사항 보고

5) 의뢰자로부터 제출된 분기별 안전성 관련 정보 보고 등

6) 이미 시판 중인 약제에서 보고된 이상약물/의료기기 반응보고 등

4. 안전성 관련 정보 보고 기한

1) 책임연구자는 이상약물/의료기기반응보고범위 이외의 이상반응 및 기타 안전성과 관련된 모든 정보에 대해서는 안전성 관련 정보보고서를 통해 보고하여야 한다.

① 개별 건이 아닌 축적된 데이터 요약본을 정기보고주기에 따라 보고하여야 한다. (예: 중간보고주기가 3개월이라면 3개월간의 안전성 관련 정보를 모아 “안전성관련정보보고서”를 작성하여 보고한다.)

② 추가적인 정보가 있는 경우에는 해당 이상약물반응이 종결(이상약물반응이 사라지거나 추적조사가 불가능하게 되는 것을 말함)될 때까지 보고하여야 한다.

5. 중증도와 인과 관계에 대한 정의.

1) 중증도

이상반응은 아래의 정의에 따라 경증, 중등증, 중증으로 구분된다.

경증은 일반적이고 일시적이고 일상적인 활동을 방해하지 않는다.

중등증은 약간의 불편함을 초래하거나 일상적인 활동을 방해한다.

중증은 일상적인 활동을 수행할 수 없다.

2) 인과 관계

인과 관계는 ‘관련 없을 것으로 생각됨’, ‘관련 있을 가능성 있음’, ’가능성 많음’, ’명백히 관련 있음’, 또는 ‘관련성을 확인하기 어려움’으로 구분된다.

**10) 자료안전성 모니터링 계획(DSMP)**

본 연구의 자료는 피험자의 권리와 복지를 보호하기 위하여 모든 피험자의 자료를 문헌으로 작성된 증례기록지 형식 혹은 암호화된 전자 파일로 작성하여 정해진 저장소에 보관한다. 본 연구는 GCP guideline에 의거하여 연구와 관계가 없는 감독관이 증례 기록지에 기록된 정보의 정확성과 완전성 그리고 검증 가능성을 확인하게 된다. 연구에 참여하는 모든 환자는 설명에 근거한 동의를 문헌으로 작성한 후 연구에 참여하게 된다.

**11) 자료 분석 및 통계 분석 방법**

본 연구에서는 두 군 간의 수축기 혈압, BIS값 데이터를 비교하기 위해 performance measurement (PM)의 방법을 시행하여 4개의 변수값을 계산 후 두 군간에 이 4 변수에 대한 군간 비교를 시행한다. PM은 원래 목표농도주입법에 의한 전정맥마취시 정맥주입용 펌프의 기계적인 적절성 또는 펌프에 사용된 약동학적 모델이나 약제의 주입 조절 알고리듬의 적절성을 후평가하기 위해 고안된 방법이다 (참고문헌 3). 그러나 이 방법은 그 적용 방법을 확대시키면 특정한 처치에 대한 환자의 반응을 평가하기 위해서 사용될 수도 있다 (참고문헌 4). PM의 계산은, 먼저 치료의 목표치와 실제 계측치로부터 performance error (PE)를 계산해 내고 이로부터 1) median performance error (MDPE), 2) median absolute performance error (MDAPE), 3) wobble, 4) divergence를 계산한다. 이러한 값들이 의미하는 바는 각각 목표한 값과 실제 값과의 1) bias 2) accuracy 와, 이러한 값들의 시간의 흐름에 따른 3) stability 4) improvement 이다.

수축기 혈압에서의 PM 계산의 예를 들면 다음과 같다.

PE*ij* = $\frac{\mathrm{SAPm}ij-\mathrm{SAPt}ij}{\mathrm{SAPt}ij}$ x 100

MDPE*i* = median{PE*ij*, *j*=1,…,N*i*}

MDAPE*i* = median{|PE*ij*|, *j*=1,…,N*i*}

Wobble*i* = median{|PE*ij*-MDPE*i*|, *j*=1,…,N*i*}

Divergence = slope{|PE*ij*|, *j*=1,…,N*i*}

SAP=systolic arterial pressure 수축기 혈압; m=measured 측정치; t=target 여기서는 100; MDPE=median PE, MDAPE=median absolute PE, slope=slope of the regression curve of |PE|’s

본 연구는 ITT 프로토콜에 따라 참여한 모든 환자(48명)에 대한 분석을 하는 보수적 접근을 하도록 한다. 통계 프로그램은 SPSS 21.0와 Microsoft Excel 2010을 사용하며 군간 평균 비교 및 분율 비교는 모두 비모수적 방법을 이용하여 시행한다. *P* < 0.05를 통계적으로 유의하다고 간주한다.

**12) 연구수행일정표**

IRB 승인일로부터 12개월

매주 평균 20건의 전신마취 하 수술에서 데이터 수집이 가능하다고 가정하는 경우 48건의 데이터 수집을 위하여 약 6개월의 시간이 요구된다. 보충 연구 및 통계 처리에 6개월의 시간을 산정하여 IRB 승인 후 12개월의 연구 기간을 예상한다.

1. **연구대상자의 안전보호를 위한 대책**
2. **연구의 윤리성 확보를 위한 기본 방안**

2013년 헬싱키 선언에 입각하여, 피험자 또는 보호자에게 연구의 목적과 연구 참여 중 일어날 수 있는 정신적, 신체적 위해를 충분히 설명한 후 피험자 (또는 보호자) 로부터 서면동의서를 받을 예정이다. 피험자, 피험자의 담당의사, 시험 참여자 이외에는 피험자의 시험 참여 여부나 치료 경과에 대해 알지 못하게 하며, 피험자의 신원을 파악할 수 있는 기록은 비밀로 보장될 것이다. 수집되는 자료의 불필요한 개인식별자는 제거하고, 특히, 증례 기록서에는 환자의 이름, 주민등록번호, 차트 번호 등을 기재하지 않도록 하며, 신상정보와 연결된 식별자 코드는 별도로 관리할 것이다. 환자와 관련된 사진을 제출할 때는 환자의 신원을 알 수 없도록 할 것이며 조금이라도 신원이 노출될 가능성이 있는 경우에는 이에 대한 서면 동의를 받았음을 명시할 것이다. 피험자의 검진 기록은 비밀이 유지되고 다른 곳으로 이동되지 않을 것이며, 본 연구의 진행 여부를 감독 받기 위해 감독 기관으로 보내어질 수 있다. 피험자의 검진 기록은 비밀이 유지되고 다른 곳으로 이동되지 않을 것이며, 본 연구의 진행 여부를 감독 받기 위해 감독 기관으로 보내어질 수 있다. 연구를 위해 수집되는 데이터는 연구 종료 후 3년 동안 보관한다. 보관 기간이 지난 문서는 개인정보 보호법 시행령 제 16조에 의하여 파기한다. 생명윤리 및 안전에 관한 법률에 따라 보관한다. 본 연구는 병원윤리위원회의 윤리규정과 ICH-GCP를 준수할 것이다.

1. **연구대상자의 동의 과정**

별첨된 설명문과 동의서에 따라 연구 담당자가 환자에게 설명한 후 서면 동의를 받는다. 동의를 제공할 자는 연구 대상자 본인과 배우자, 자녀 등의 직계 대리인으로 한정한다. 연구 설명 후 환자가 자발적으로 동의할 때까지 필요한 충분한 시간을 준다. 연구 동의 과정에는 어떠한 강제성도 없을 것이며 연구 설명은 일반인이 받아들이기 쉬운 평이한 언어로 시행할 것이다. 연구 동의를 받은 후 설명문 사본 한 부를 환자가 보관하도록 한다.

1. **연구대상자의 보상 방안**

해당사항 없음

1. **연구대상자의 개인정보보호 방안**

피험자의 신원을 파악할 수 있는 기록은 비밀로 보장될 것이다. 수집되는 자료의 불필요한 개인식별자는 제거하고, 특히 증례 기록서에는 환자의 이름, 주민등록번호, 차트 번호 등을 기재하지 않도록 하며 신상정보와 연결된 식별자 코드는 별도로 관리할 것이다. 전자 파일의 경우 암호화하여 정해진 저장소에만 보관한다. 환자와 관련된 사진을 제출할 때는 환자의 신원을 알 수 없도록 할 것이며 조금이라도 신원이 노출될 가능성이 있는 경우에는 이에 대한 서면 동의를 받았음을 명시할 것이다. 피험자의 검진 기록은 비밀이 유지되고 다른 곳으로 이동되지 않을 것이며, 본 연구의 진행 여부를 감독 받기 위해 감독 기관으로 보내어질 수 있다. 피험자의 검진 기록은 비밀이 유지되고 다른 곳으로 이동되지 않을 것이며, 본 연구의 진행 여부를 감독 받기 위해 감독 기관으로 보내어질 수 있다. 연구를 위해 수집되는 데이터는 연구 종료 후 3년 동안 보관한다.

1. **취약한 연구대상자를 포함하는 경우 추가적인 보호조치 방안**

해당사항 없음

1. **인체유래물의 보관 및 폐기 방법**

해당사항 없음

1. **참고 문헌**
2. Manyam SC, Gupta DK, Johnson KB, White JL, Pace NL, Westenskow DR, et al. Opioid–Volatile Anesthetic Synergy: A Response Surface Model with Remifentanil and Sevoflurane as Prototypes. Anesthesiology. 2006;105(2):267-78.
3. Avidan MS, Zhang L, Burnside BA, Finkel KJ, Searleman AC, Selvidge JA, Saager L, Turner MS, Rao S, Bottros M, Hantler C, Jacobsohn E, Evers AS. Anesthesia awareness and the bispectral index. N Engl J Med. 2008 Mar 13;358(11):1097-108.
4. Varvel JR, Donoho DL, Shafer SL. Measuring the predictive performance of computer-controlled infusion pumps. J Pharmacokinet Biopharm. 1992;20(1):63-94.
5. Struys MM, De Smet T, Versichelen LF, Van De Velde S, Van den Broecke R, Mortier EP. Comparison of closed-loop controlled administration of propofol using Bispectral Index as the controlled variable versus "standard practice" controlled administration. Anesthesiology. 2001;95(1):6-17.
6. Pollard RJ, Coyle JP, Gilbert RL, Beck JE. Intraoperative awareness in a regional medical system: a review of 3 years' data. Anesthesiology. 2007;106(2):269-74.

**[별첨1] 연구대상자 설명문 및 동의서**

**1. 임상시험 제목**

데스플루란-레미펜타닐 균형 마취에서 데스플루란 투여 방법 비교: Bispectral Index (BIS)에 의한 조절법 대 고정 농도 주입법

**2. 연구책임자**

서울대학교병원 마취통증의학과 교수 정철우

**3. 임상시험의 배경 및 목적**

본 연구는 균형 마취에서 흡입 마취제를 조절하는 두 가지 방식을 비교하기 위한 연구입니다. 균형 마취란 현대 마취에서 널리 이용되는 방식으로서, 흡입 마취제에 적정량의 아편 유사제를 함께 사용하여 혈역학적으로 안정적인 마취 유지와 수술 종료 후 빠른 각성을 도모할 수 있습니다.

BIS (Bispectral Index)란 환자분의 마취 중 각성 상태를 나타내는 감시 장치이며 전신 마취 중에는보통 40-60 사이로 유지합니다. 본 연구에서는 데스플루란과 레미펜타닐을 함께 사용하는 균형 마취에서 환자의 흡입 마취제를 BIS 값 50을 목표로 조절하는 것과 흡입 마취제의 농도를 일정량으로 고정하여 사용하는 방법의 임상적인 효용성과 안정성을 비교하고자 합니다. 균형 마취를 시행할 때 임상의의 판단에 따라 흡입 마취제의 농도를 특정값에 고정하여 유지하는 방법과 BIS를 50을 목표로 조절하는 방법 중 어떤 것이 임상적으로 우월하다고 검증된 바 없으며, 따라서 본 연구를 시행하고자 합니다.

**4. 임상시험용 의약품/의료기기**

해당사항 없음

**5. 임상시험 참여대상자 수 및 참여기간**

전신 마취하 수술을 받기로 예정된 대상자에게 시험 참여를 요청하며 본 연구에는 48명이 참여할 예정입니다. 각 군에 배정되는 비율은 동일합니다. 예상 참여 기간은 마취 시작부터 수술 다음날까지입니다.

**6. 임상시험 방법**

귀하는 전신 마취를 위해 흡입 마취제 데스플루란과 아편 유사제 레미펜타닐을 사용하게 되며, 마취 중 각성 정도를 감시받기 위해 BIS 감시 장치를 부착하게 됩니다. 본 연구에 참여하시면 무작위로 두 군(데스플루란의 농도를 BIS 값 50을 목표로 조절하는 군, 흡입 마취제인 데스플루란의 농도를 일정량으로 고정하여 사용하는 군) 중 한 군으로 지정됩니다. 군 배정은 마취 시작 전 무작위로 배정되므로 어느 군에 속할지는 미리 알 수 없으며, 귀하가 두 군중 하나를 임의로 선택하실 수는 없습니다. 전신 마취는 BIS를 이용한 각성도 감시 하에 안전한 수준에서 진행되며 귀하의 생체 징후 역시 지속적으로 감시합니다. 마취 중 귀하의 혈압, 맥박수, BIS 값을 수집하여 연구에 이용하게 됩니다. 수술 종료 후 마취제 종료 시점부터 귀하가 가벼운 자극(음성 자극, 가벼운 두드림)에 대해 눈을 뜬 시간이 기록됩니다. 수술 다음날 귀하의 수술 중 각성 여부를 판단하기 위한 면담이 진행됩니다. 발생할 수 있는 부작용이나 합병증과 그 이유가 기록될 것입니다.

**7. 대안 치료 (임상시험 이외의 다른 대체 가능한 치료법)**

연구에 참여하지 않는 경우에도 단지 데이터의 수집을 하지 않을 뿐 균형마취의 방법이 여전히 적용될 수 있습니다. 균형마취를 원하지 않을 경우에는 레미펜타닐의 지속정주를 병용하지 않는 흡입마취 단독 사용법 또는 정맥마취의 방법으로 마취가 진행됩니다.

**8. 연구대상자에게 예견되는 부작용, 위험과 불편함**

전신마취에 따른 부작용과 위험이 본 연구에서 동일하게 발생할 수 있으며 이는 저혈압, 서맥, 그리고 매우 낮은 빈도의 수술중 각성 등을 포함합니다. 그러나 본 연구에서의 마취 방법인 데스플루란과 레미펜타닐을 함께 사용하는 균형 마취의 방법은 이미 널리 사용되는 전신 마취 방법으로서, 환자가 본 연구에 참여시 기존의 전신 마취에서 보고된 위험에 비해 추가적인 위험에 노출되지는 않습니다. 두 군 모두 흡입 마취제의 농도는 임상적으로 안전한 마취 농도에서 BIS를 이용한 각성도 감시 하에 이루어지며 레미펜타닐 주입량도 모두 임상적으로 사용되는 범위 내에서 이루어집니다.

**9. 연구대상자에게 예견되는 이득**

일반적인 흡입 마취제만을 이용한 전신 마취의 경우에 비해 균형 마취를 시행할 경우 혈역학적으로 안정적인 마취 유지와 수술 종료 후 빠른 각성이 가능합니다. 본 연구의 결과가 나왔을 때, 이를 학문적으로 학계에 보고하여 결과를 향상시키는데 도움을 줄 수 있다면, 이로 인해 수많은 환자들에게 긍정적인 영향을 줄 수 있을 것으로 생각됩니다.

**10. 비용 및 보상**

연구에 따른 보상은 없습니다.

**11. 자발적 참여/연구의 중단**

연구의 참여는 본인의 자발적인 의사에 의해서만 가능합니다. 만일 참여를 원하지 않으신다고 하여도 추후의 치료과정에는 어떤 영향도 미치지 않을 것입니다. 대상자께서 원하신다면 시험 도중 언제라도 중도에 참여를 포기할 수 있으며, 추후 치료과정에는 어떠한 영향도 없을 것입니다. 또한 시험 도중 피험자의 참여의지에 영향을 줄 수 있는 새로운 정보가 수집된다면 즉시 피험자 또는 대리인에게 알릴 것입니다 임상 시험 도중 대상자의 임상 시험 참여가 중지된다면 그 사유가 기록됩니다.

**12. 연구 관련 새로운 정보의 지속적 제공**

연구 참여 도중 임상시험 참여 여부에 영향을 줄 수 있는 새로운 정보는 귀하 혹은 귀하의 대리인에게 고지됩니다.

**13. 손상 및 보상**

연구에 따른 추가적인 위험은 없으므로 이에 대한 특별 보상은 없으나, 전신 마취 시행에 따른 손상이 발생시 병원의 통상적인 보상 규정에 따른 보상이 이루어집니다.

**14. 비밀 보장**

본 연구 결과는 학술 목적으로만 이용되며 학술적인 보고를 위하여 외부로 발표될 수 있습니다. 대상자와 대상자의 담당의사, 그리고 시험 참여자 이외에는 대상자의 시험 참여 여부나 치료 경과에 대해서는 알지 못할 것입니다. 또한 피험자의 신원을 파악할 수 있는 기록은 비밀로 보장될 것이며, 임상시험의 결과가 출판될 경우 피험자의 신원은 비밀상태로 유지될 것입니다. 대상자의 검진 기록은 비밀이 유지되고 다른 곳으로 이동되지 않을 것이며, 본 연구의 진행 여부를 감독 받기 위해 감독 기관으로 보내어 질 수 있습니다.

대상자께서 동의하신다면 진료를 위해 다른 의사에게 시험의 참여 사실이 통보될 것입니다. 귀하의 기록은 법이 정하는 기간 동안 보관될 것이며 추후 모든 자료는 폐기될 것입니다.

모니터 요원, 점검을 실시하는 사람, 심사위원회 및 식품의약품안전처장이 관계 법령에 따라 임상 시험의 실시 절차와 자료의 품질을 검증하기 위하여 대상자의 신상에 관한 비밀이 보호되는 범위에서 대상자의 의무 기록을 열람할 수 있으며 대상자 또는 대상자의 대리인이 서명한 동의서에 의하여 이러한 자료의 열람이 허용됩니다.

**15. 담당자 연락처**

임상시험 참여와 관련하여 귀하의 권익에 관한 추가적인 정보를 얻고자 하는 경우, 서울대학교병원 의학연구윤리심의위원회 (02-2072-0694)로 문의하여 주시기 바랍니다.

임상 시험과 관련한 의문이나 손상이 발생한 경우 다음 연락처로 연락하시기 바랍니다.

연구책임자: 서울대학교병원 마취통증의학과 교수 정철우 02-2072-0640

연구담당자: 서울대학교병원 마취통증의학과 전임의 정유선 02-2072-7361

**동의서**

데스플루란-레미펜타닐 균형 마취에서 데스플루란 투여 방법 비교: Bispectral Index (BIS)에 의한 조절법 대 고정 농도 주입법

1. 본인은 임상시험에 대해 구두로 설명을 받고 상기 연구 설명문을 읽었으며 연구 담당자와 이 연구에 대하여 충분히 의논하였습니다.

2. 본인은 연구의 위험과 이득에 관하여 들었으며 나의 질문에 만족할 만한 답변을 얻었습니다.

3. 본인은 이 연구에 참여하는 것에 대하여 자발적으로 동의합니다.

4. 본인은 이후의 치료에 영향을 받지 않고 언제든지 연구의 참여를 거부하거나 연구의 참여를 중도에 철회할 수 있고 이러한 결정이 나에게 어떠한 해가 되지 않을 것이라는 것을 알고 있습니다.

5. 본인은 이 설명서 및 동의서에 서명함으로써 의학 연구 목적으로 나의 개인정보가 현행 법률과 규정이 허용하는 범위 내에서 연구자가 수집하고 처리하는데 동의합니다.

6. 본인은 연구 설명문 및 동의서의 사본을 받을 것을 알고 있습니다.

|  |  |  |  |  |
| --- | --- | --- | --- | --- |
| 연구대상자 성명 |  | 서명 |  | 날짜(년/월/일) |
|  |  |  |  |  |
| 시험자/연구자 성명 |  | 서명 |  | 날짜(년/월/일) |
|  |  |  |  |  |
| 법정대리인 성명 |  | 서명 |  | 날짜(년/월/일) |
|  |  |  |  |  |
| (대상자와 대리인과의 관계) |  |  |  |  |

**[별첨2] 증례기록서**

**Case Report Form (Serial No._____)**

Recorder ____________________

| Bispectral Index-Guided versus Fixed Dose Administration of Desflurane during Balanced Anesthesia with Remifentanil |
| --- |

| **날짜** |  | |  |  | | **연구자 서명** | |  |  |
| --- | --- | --- | --- | --- | --- | --- | --- | --- | --- |
|  |  | |  |  | |  | |  |  |
| Op name |  | |  |  | |  | |  |  |
|  |  | |  |  | |  | |  |  |
| Group | BIS-guided / Fixed | | |  | | Serial No | |  |  |
|  |  | |  |  | |  | |  |  |
| Age/Sex | / | |  |  | | Ht/Wt | | / |  |
|  |  | |  |  | |  | |  |  |
| Anes time |  | | min |  | | Op time | |  | min |
|  |  | |  |  | |  | |  |  |
| Time to awake | sec | | |  | |  | |  |  |
|  |  | |  |  | |  | |  |  |
| **Use of drugs** |  | |  |  | |  | |  |  |
| Remifentanil |  | | mcg |  | |  | |  |  |
| Desflurane |  | | ml |  | |  | |  |  |
| Muscle Relaxant | mg | | |  | |  | |  |  |
|  |  |  | | |  | | Change of concentration | | |
| Intraoperative awareness |  | | | | Remifentanil | |  | | |
|  |  |  |  |  | Desflurane | |  | | |
|  |  |  | | |  | |  | | |
| *BIS, Systolic arterial pressure, Heart rate는 환자 모니터로부터 시리얼 포트를 통해 직접 다운로드하여 환자의 일련번호와 연동하여 암호화된 전자 파일로 보관 | | | | | | | | | |

| 이상 반응 평가 | | | | |
| --- | --- | --- | --- | --- |
| 이상 반응 | 중등도 | 기간 | 처치 | 인과관계 |
|  |  |  |  |  |
|  |  |  |  |  |

**Interview Form (Serial No._____)**

Recorder ____________________

| Bispectral Index-Guided versus Fixed Dose Administration of Desflurane during Balanced Anesthesia with Remifentanil |
| --- |

| **작성 날짜** |  |  | **연구자 서명** |  |
| --- | --- | --- | --- | --- |
|  | | | | |
| 1. 수술 전 마지막으로 기억나는 것은 무엇입니까? | | | | |
|  | | | | |
| 2. 깨어나신 후 처음으로 기억나는 것은 무엇입니까? | | | | |
|  | | | | |
| 3. 수술 중 꿈을 꾸었습니까? | | | | |
|  | | | | |
| 4. 부드럽게 잠이 들었습니까? | | | | |
|  | | | | |
| 5. 잠이 들기까지의 과정에 문제점은 없었습니까? | | | | |
|  | | | | |

| 이상 반응 평가 | | | | |
| --- | --- | --- | --- | --- |
| 이상 반응 | 중등도 | 기간 | 처치 | 인과관계 |
|  |  |  |  |  |
|  |  |  |  |  |

**[별첨4] 임상시험 피해자 보상규약**

1. 본 임상시험의 연구자는 임상시험기간 동안 합의된 임상시험계획서에 따라 시행된 과정의 결과로 피험자에게 피해가 발생하였을 경우 책임을 진다. 단, 연구자가 책임을 지는 경우는 피험자에게 발생하는 피해와 임상연구 사이에 인과관계가 없음을 입증하지 못하는 경우로 제한된다.

2. 다음의 경우에는 보상 대상에서 제외된다.

- 임상 연구와 관련이 없이 발생한 부작용

- 연구와는 무관하게 피험자가 원래부터 가지고 있었던 기저질환의 악화

- 피험자의 부주의에서 초래된 손상

- 임상 실험 도중 대상에서 제외되어 연구가 중단된 이후 발생한, 연구와 직접적인 관련이 없이 발생한 손상

3. 임상시험 책임자는 피험자가 본 임상시험에 의해 어떠한 불이익이라도 받지 않도록 관계법규와 규범, 상호 합의한 임상시험계획서의 내용을 충실히 준수하는 등 최선을 다해야 한다. 그러나 이러한 노력에도 불구하고 연구자가 시행한 임상 시험에 의해 피험자가 피해(상해 또는 부작용 발생)를 입게 된 경우는 아래의 보상평가기준에 따라 합리적인 치료비 또는 보상금을 지급한다.

[보상평가 기준]

치료비 또는 보상금은 피해의 본질, 정도, 기간, 지속성여부 등에 따라 이를 치료 또는 보상할 수 있는 적절한 액수여야 하며 한국법정에서 유사피해에 대해 일반적으로 지급되도록 하는 것과 동일한 수준으로 한다. 보상수준에 대해 피험자와 연구자 사이에 이견이 있을 경우에는 우선적으로 양 당사자의 합의 하에 선정한 전문가로부터 자문을 구하도록 하고, 자문을 받을 경우에는 자문내용에 따르기로 한다.

본 임상시험에서 피험자가 불이익을 받지 않도록 주의를 기울이고, 피험자가 입는 피해에 대하여 상기 내용에 의거하여 책임질 것을 서약합니다.

2014. 06. 연구 책임자

서울대학교병원 마취통증의학과

교수 정 철 우


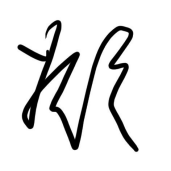
**[별첨5] 책임연구자의 이력**

**학력**

1995년 서울대학교 의과대학 졸업

2000년 서울대학교 대학원 의학 석사 (마취과학 전공)

2005년 서울대학교 대학원 의학 박사 (마취과학 전공)

**자격**

1995년 의사면허증 취득 (의사 면허 번호 55828)

2000년 마취과 전문의 자격증 취득 (전문의 번호 2183)

**경력**

1995년 3월 – 1996년 2월 서울대학교 병원 수련의

1996년 3월 – 2000년 2월 서울대학교 병원 마취과 전공의

2001년 4월 – 2003년 4월 국군 서울 지구 병원 마취과장 (해군 대위)

2003년 5월 – 2004년 4월 서울대학교 병원 마취통증의학과 전임의

2004년 5월 – 2005년 4월 서울대학교 병원 마취통증의학 촉탁의

2005년 5월 – 2005년 2월 서울대학교 병원 마취통증의학 임상교수

2009년 3월 – 2013년 2월 서울대학교 마취통증의학과 조교수

2013년 3월 – 현재 서울대학교 마취통증의학과 부교수

**GCP 교육 수료 여부**

2013.10.1. 다음 교육을 이수함.

- GCP and clinical research : overview

- GCP : Investigator's role and responsibilities

- GCP : IRB's role and responsibilities

**저서**

Ho-Geol Ryu, Chul-Woo Jung, Hyung-Chul Lee, and Youn-Joung Cho

Epinephrine and Phenylephrine Pretreatments for Preventing Postreperfusion Syndrome During Adult Liver Transplantation

LIVER TRANSPLANTATION 2012 DEC 18:1430-1439

Lim T, Ryu HG, Jung CW, Jeon Y, Bahk JH.

Effect of the bevel direction of puncture needle on success rate and complications during internal jugular vein catheterization.

Crit Care Med. 2012 Feb;40(2):491-4.

Ryu HG, Jung CW, Lee CS, Lee J.

Nafamostat Mesilate Attenuates Postreperfusion Syndrome during Liver Transplantation.

Am J Transplant. 2011 May;11(5):977-83

Lee KH, Nam SH, Yoo SY, Jung CW, Bae SS, Lee JR.

Vecuronium requirement during liver transplantation under sevoflurane anesthesia.

J Anesth. 2010 Jul 6.

Ryu HG, Nahm FS, Sohn HM, Jeong EJ, Jung CW.

Low central venous pressure with milrinone during living donor hepatectomy.

Am J Transplant. 2010 Apr;10(4):877-82.

Lee JH, Bahk JH, Ryu HG, Jung CW, Jeon Y.

Comparison of the bedside central venous catheter placement techniques: landmark vs electrocardiogram guidance.

Br J Anaesth. 2009 May; 102(5): 662-6

Lee JR, Jung CW*, Lee YH.

Reduction of pain during induction with target-controlled propofol and remifentanil.

Br J Anaesth. 2007 Dec;99(6):876-880.

Seo JH, Jung CW, Bahk JH*

Uppermost blood levels of the right and left atria in the supine position: implication for measuring central venous pressure and pulmonary artery wedge pressure.

Anesthesiology. 2007 Aug;107(2):260-3.

Jung CW, Seo JH, Lee W, Bahk JH*

A novel supraclavicular approach to the right subclavian vein based on three-dimensional computed tomography.

Anesth Analg. 2007 Jul;105(1):200-4.

Jung CW, Kim JT, Lee KH*

The hemodynamic effects of insulin following overdosage with levobupivacaine or racemic bupivacaine in dogs.

J Korean Med Sci. 2007 Apr;22(2):342-6.

Hyo-Jin Byon, Keun-Suk Park, Yong-Hee Park, Jin-Tae Kim, Chul-Woo Jung and Hee-Soo Kim*

The influence of DNA polymorphism of multidrug resistant 1 (MDR1) on the effect of midazolam pretreatment in children

Korean J Anesthesiol. 2012 Apr;62(4):332-336.

Tae Dong Kweon, Chul-Woo Jung, Jin-Woo Park, Yun-Seok Jeon, and Jae-Hyon Bahk*

Hemodynamic effect of full flexion of the hips and knees in the supine position: a comparison with straight leg raising

Korean J Anesthesiol. 2012 Apr;62(4):317-321

Ji-Won Lee, Chul-Woo Jung

The target concentration of remifentanil to suppress the hemodynamic response to endotracheal intubation during inhalational induction with desflurane

Korean Journal of Anesthesiology 2011 Jan; 60(1): 12-18

이정림, 정철우*, 홍덕만

간이식 수혜자에서 내경정맥 도관술 후 발생한 동정맥루관 ⁣증례보고⁣

대한마취과학회지 2007 Apr; 052(04): 487-490.

Kim JT, Shim JK, Kim SH, Jung CW, Bahk JH*

Trendelenburg position with hip flexion as a rescue strategy to increase spinal anaesthetic level after spinal block.

Br J Anaesth. 2007 Mar;98(3):396-400.

서정화,이 활,정철우*

삼차원 단층촬영을 이용한 중심정맥의 쇄골상부 도자법에 대한 해부학적 고찰

대한마취과학회지 2006 Apr; 050(04): 373-378.

구미숙, 정철우*

근이완제에 교차감작된 환자에서 복강경 수술의 마취 경험 - 증례보고 -

대한마취과학회지 2006 Jan; 050(01): 90-93

Ahn W*, Jung CW.

A comparison of the Vasotrac with invasive arterial blood pressure monitoring.

Anesth Analg. 2006 Jan;102(1):333.

이정림, 정철우*, 이종환, 최인용, 서광석, 김혜경, 도상환, 김종성

후두경을 이용한 기관내 삽관시의 심혈관계 반응을 억제하는 Remifentanil의 적절한 용량

대한마취과학회지 2005 Dec; 049(06): 780-785

배영곤, 정철우, 김종성*

방사성 옥소(I-131) 치료환자의 응급 수술시 발생한 방사선 피폭 - 증례보고 -

대한마취과학회지 2005 Nov; 049(05): 739-743.

Kim JT, Jung JY, Jung CW, Kim JA, Cho HS, Lee KH*

S-wave in lead III is helpful for the early detection of bupivacaine-induced cardiac depression in dogs: [L'onde S en D III est utile pour la detection precoce de la depression myocardique induite par la bupivacaine chez les chiens].

Can J Anaesth. 2005 Oct;52(8):864-9.

정철우, 이미금, 이국현*

간이식 수술시 발생한 전격성 폐부종의 치료를 위한 응급 정맥-동맥 우회술의 이용

대한마취과학회지 2005 Sep; 049(03): 417-420.

Ho-Geol Ryu, M.D., Chul-Woo Jung, M.D., Jahng-Hyon Park, Ph.D.*, Young-Jun Um, M.D., and Jae-Hyon Bahk, M.D*

The Resting Volume of the Bronchial Cuff of the Left-sided Double-lumen Tube and the Diameter of the Left Mainstem Bronchus Indicated for Each Double-lumen Tube Size

대한마취과학회지 2005; 48: S 1∼4

Kwon TD, Kim KH, Ryu HG, Jung CW, Goo JM, Bahk JH*

Intra- and extra-pericardial lengths of the superior vena cava in vivo: implication for the positioning of central venous catheters.

Anaesth Intensive Care. 2005 Jun;33(3):384-7.

Jeon Y, Ryu HG, Bahk JH*, Jung CW, Goo JM

A new technique to determine the size of double-lumen endobronchial tubes by the two perpendicularly measured bronchial diameters.

Anaesth Intensive Care. 2005 Feb;33(1):59-63.

정철우, 김진태, 최윤숙, 배성심, 김지애, 조현성, 이국현*

부피바케인과 레보부피바케인으로 심장혈관허탈을 유도한 개에서 혈류역학의 비교

대한중환자의학회지 2004 Dec; 19(02): 86-97

Kim JT, Jung CW, Lee KH*

The effect of insulin on the resuscitation of bupivacaine-induced severe cardiovascular toxicity in dogs.

Anesth Analg. 2004 Sep;99(3):728-33

허용준, 정철우, 조주연, 김용락*

자가통증조절법의 시작 시점이 술 후 진통효과 발현 시간에 미치는 영향

대한마취과학회지 2004 Jul; 47(1): 101-105

Jung CW, Bahk JH*, Lee JH, Lim YJ

The tenth rib line as a new landmark of the lumbar vertebral level during spinal block.

Anaesthesia. 2004 Apr;59(4):359-63

Kim JT, Jung CW, Lee JR, Min SW, Bahk JH*

Influence of lumbar flexion on the position of the intercrestal line.

Reg Anesth Pain Med. 2003 Nov-Dec; 28(6): 509-11

정철우·안원식*

한국 성인 남자에서 정중접근법에 의한 척추마취시 최적의 삽입점과 삽입각에 관한 연구

대한마취과학회지 2003 Apr; 044(04): 494-499

Jung CW, Bahk JH*, Kim MW, Lee KH, Ko H

Head position for facilitating the superior vena caval placement of catheters during right subclavian approach in children.

Crit Care Med. 2002 Feb; 30(2): 297-9

정철우, 김지연, 박재현, 고 홍*

소아에서 쇄골하정맥 도자시 도관의 상대정맥내 거치율을 증가시키는 방법

대한마취과학회지 2000 Jun; 038(06):

정철우, 임영진*, 김성덕

소아에서 쇄골하정맥 도자시 도관의 적정 삽입 깊이

대한마취과학회지 1999 Sep; 037(03):

Bahk JH*, Jung CW, Kim SD

A guide for tube exchange using a fibrescope and the plastic sheath of a guidewire in small children.

Br J Anaesth. 1998 Jul; 81(1): 103

심지연, 정철우, 고 홍*

고관절 전치환술 환자에서 시행한 급성 동량성 혈액희석시의 Thrombelastography의 변화

대한마취과학회지 1998 Jun; 034(06)
